# An evolutionarily diverged mitochondrial protein controls biofilm growth and virulence in *Candida albicans*

**DOI:** 10.1371/journal.pbio.3000957

**Published:** 2021-03-15

**Authors:** Zeinab Mamouei, Shakti Singh, Bernard Lemire, Yiyou Gu, Abdullah Alqarihi, Sunna Nabeela, Dongmei Li, Ashraf Ibrahim, Priya Uppuluri

**Affiliations:** 1 David Geffen School of Medicine, University of California (UCLA), Los Angeles, California, United States of America; 2 Division of Infectious Disease, The Lundquist Institute for Biomedical Innovation at Harbor-UCLA Medical Center, Torrance, California, United States of America; 3 Department of Biochemistry, University of Alberta, Alberta, Canada; 4 Department of Microbiology and Immunology, Georgetown University Medical Center, Washington, DC, United States of America; University of California, San Francisco, UNITED STATES

## Abstract

A forward genetic screening approach identified orf19.2500 as a gene controlling *Candida albicans* biofilm dispersal and biofilm detachment. Three-dimensional (3D) protein modeling and bioinformatics revealed that orf19.2500 is a conserved mitochondrial protein, structurally similar to, but functionally diverged from, the squalene/phytoene synthases family. The *C*. *albicans* orf19.2500 is distinguished by 3 evolutionarily acquired stretches of amino acid inserts, absent from all other eukaryotes except a small number of ascomycete fungi. Biochemical assays showed that orf19.2500 is required for the assembly and activity of the NADH ubiquinone oxidoreductase Complex I (CI) of the respiratory electron transport chain (ETC) and was thereby named *NDU1*. *NDU1* is essential for respiration and growth on alternative carbon sources, important for immune evasion, required for virulence in a mouse model of hematogenously disseminated candidiasis, and for potentiating resistance to antifungal drugs. Our study is the first report on a protein that sets the *Candida*-like fungi phylogenetically apart from all other eukaryotes, based solely on evolutionary “gain” of new amino acid inserts that are also the functional hub of the protein.

## Introduction

*Candida albicans* biofilms are dynamic communities in which transitions between planktonic and sessile modes of growth occur interchangeably in response to different environmental cues. Biofilms growing on mucosal tissues or indwelling medical devices serve as localized reservoirs of highly drug resistant cells. Cells that disperse from this nidus into the systemic environment cause biofilm-associated disseminated infections [1,2]. Our previous reports have shown that biofilm-dispersed cells are predominantly lateral yeast cells released from the hyphal layers of the biofilm [3]. Phenotypically, biofilm-dispersed yeast cells have considerably better adherence to, and invasion of human tissues when compared to planktonic cells, and thereby are significantly more virulent than their free-living counterparts [3,4]. Global transcriptomic analysis of dispersed cells corroborated the virulence attributes, revealing expression of adhesins and invasins and secreted aspartyl protease genes, at levels similar to, or even statistically enhanced than parent biofilms [4]. Interestingly, it was also found that the dispersed cells are transcriptionally reprogrammed before release, to acquire nutrients such as zinc and amino acids and to metabolize alternative carbon sources, while their biofilm-associated parent cells did not induce high-affinity transporters or gluconeogenetic genes, despite exposure to the same nutritional signals [4]. Expression of genes required during starvation such as those encoding transporters, the tricarboxylic acid (TCA) cycle, and glyoxylate cycle components also implies that dispersed lateral yeast cells may have enhanced respiratory capacity over their metabolically dormant hyphal parents.

While regulatory networks governing *C*. *albicans* biofilm formation have been well defined [5], hardly anything is known about the genes/proteins controlling biofilm dispersal. To date, *C*. *albicans* protein PES1 is the only molecular regulator that has been shown to control production of lateral yeast cells from hyphae and to induce biofilm dispersal [4,6]. Thus, we embarked on a study to identify additional novel regulators of biofilm dispersal. Considering that dispersed cells are in a developmental phase distinct from the biofilm state, we hypothesized that some regulators may have a role in cellular metabolism or respiration.

Here, we report on the discovery of *NDU1*, a gene that encodes a mitochondrial protein required for the assembly and activity of the NADH ubiquinone oxidoreductase Complex I (CI) of the respiratory electron transport chain (ETC). Studies in *C*. *albicans* using gene deletion and complementation mutants revealed that *NDU1* is important for lateral yeast production and biofilm dispersal, and absence of *NDU1* triggers early biofilm detachment from its growth substrate. Our results further showed that *NDU1* is essential for respiration and growth on alternative carbon sources, potentiates resistance to antifungal drugs, is important for immune evasion and full virulence in a mouse model of hematogenously disseminated candidiasis. Importantly, NDU1 protein has diverged significantly from other eukaryotic orthologues including the human orthologue NDUFAF6 [7]; NDU1 protein harbors stretches of amino acid sequences acquired over evolution, which are uniquely specific only to *Candida*-like fungi, and can be the target for development of novel therapies.

## Results

### Loss of orf19.2500 abrogates *C*. *albicans* biofilm dispersal and induces early biofilm detachment

To identify potential regulators of biofilm dispersal, we performed forward genetic screening of several libraries of *C*. *albicans* mutants available through the Fungal Genetics Stock Center (FGSC) (Manhattan, Kansas, United States of America). The libraries encompassed disruption mutants in *C*. *albicans* genes encoding transcription factors, kinases, cell wall integrity, and hundreds of other nonessential genes. Biofilms were developed from stationary phase cultures of each mutant on the MBEC Assay plates (Innovotech, Edmonton, Canada) which enables for high-throughput screening of biofilm formation and dispersal [8]. Of all the mutant strains that grew robust biofilms, two were isolated for their significant reduction in the frequency of biofilm dispersal. One strain was a mutant of the *C*. *albicans* major phosphodiesterase gene *PDE2*. The role of *PDE2* in cAMP-mediated control of lateral yeast production from hyphae has been previously published, and hence reduced dispersal from biofilms was expected [9]. The other strain with abrogated biofilm dispersal was a mutant with deletions in an uncharacterized gene, orf19.2500.

Independent gene deletion mutants of orf19.2500 (orf19.2500−/−) were constructed using a PCR-based gene disruption approach using small homology regions. In addition, complemented strains were constructed in which both alleles of orf19.2500 were reconstituted into the mutant (orf19.2500+/+). The ability of these mutant strains to develop biofilms was assessed in the 24-well polystyrene plates incubated under static conditions or under flow of liquid medium on silicone elastomer (SE) material.

**Fig 1:**
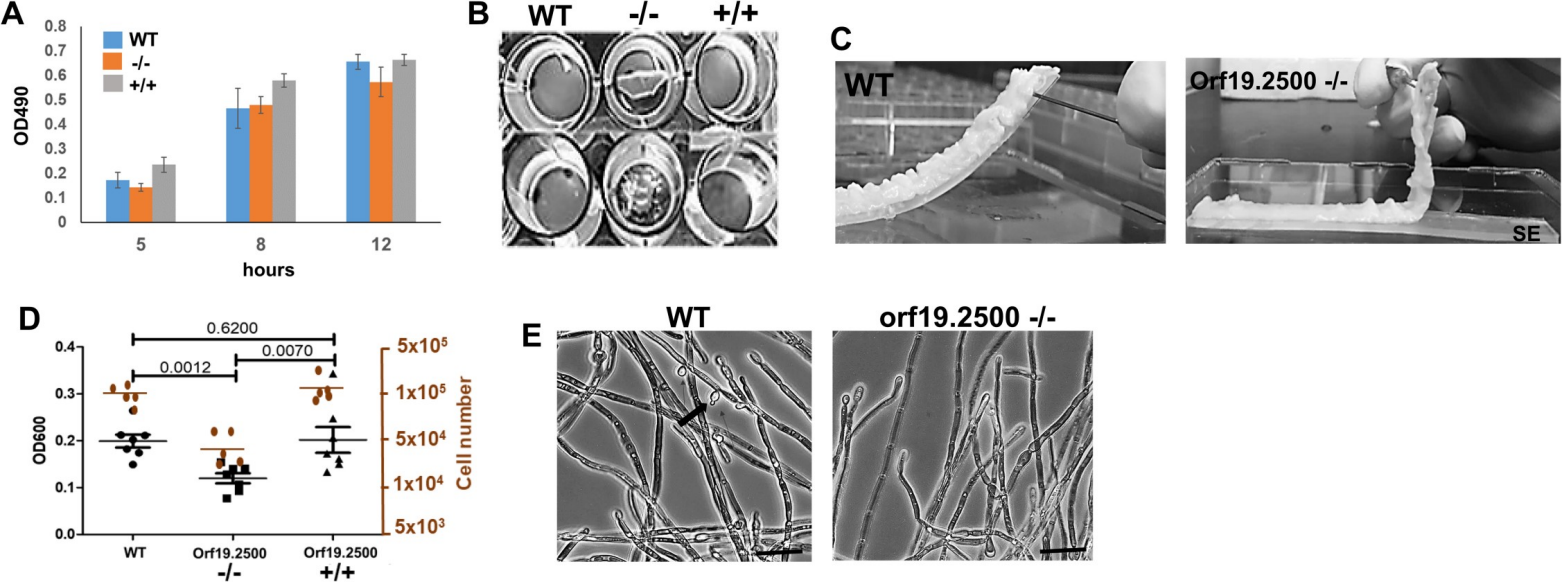
Extent of biofilm dispersal and attachment. (Note: Once its localization and function was determined, orf19.2500 was renamed as *NDU1* in later figures). *C*. *albicans* WT, orf19.2500 deletion mutant −/−, and revertant +/+ were allowed to develop biofilms using RPMI medium under static conditions for 24 h at 37°C. At various points during the biofilm growth (5 h, 8 h, and 12 h), biofilms (7 replicates each) were quantified using the XTT assay, as previously described [57, 63] (A). Biofilms of the 3 strains were developed on 96-well microtiter plates under static conditions for 24 h, after which they were gently washed once to examine the robustness of their attachment to the well surface (B). Biofilms of the WT and orf19.2500 mutant were developed overnight on the surface of SE under continuous flow of fresh RPMI at 37°C. The biofilms were gently teased away from the substrate using a needle to test the sturdiness of their attachment to the SE strips (C). Dispersal from 24-h static as well as flow biofilms were measured by OD600 density (for static, black symbols) or by counting the released cells using a hemocytometer (flow, orange dots), providing a measure of the extent of biofilm dispersal between the 3 conditions. Values are average + SEM; indicated *p*-values are measurements from 7 independent replicates of the static biofilm model. Also, dispersal from mutant biofilms under the flow conditions was significantly lower than WT or +/+ (*p* < 0.01) (D). Topmost hyphal layer of the biofilms were imaged using a light microscope (40× mag), to visualize the extent of lateral yeast growth from mutant hyphae (arrow), versus the WT. Scale bar = 20 μM (E). Raw data are found in the file S1 Data. SE, silicone elastomer; WT, wild-type.

Under static biofilm induction, both orf19.2500−/− and +/+ developed biofilms comparable to wild-type (WT) [as quantified over time, **Fig 1A,** raw data are found in the file S1 Data], but only mutant biofilms detached early (16 to 20 h of growth) (**Fig 1B**). The layer of biofilm formed by the mutant strains either peeled off completely or broke into pieces upon gentle washing of the biofilms with phosphate buffered saline. Similarly, under flow biofilm conditions, both WT and orf19.2500 −/− developed robust biofilms, but while the WT biofilms were firmly attached to SE after 24 h of growth, mutant biofilms were easily displaced from the surface (**Fig 1C**).

Supernatant media from the static model or media flowing over flow biofilms growing on SE were collected and quantified by measuring OD600 or by hemocytometer-based cell counts, respectively. Mutant biofilms displayed an overall 2- to 5-fold decrease in biofilm dispersal compared to the WT or complemented strains in both static or the flow system (**Fig 1D**, raw data are found in the file S1 Data). When the frequency of dispersal was measured over time, decrease in release of cells from the orf19.2500 mutant biofilm versus the WT or revertant biofilms was particularly apparent during logarithmic biofilm growth (12 h to 16 h) (**S1A Fig**, raw data are found in the file **S1 Data**). Considering biofilm dispersal is a consequence of lateral yeast cells shed by biofilm hyphae in the surrounding media [3], topmost hyphal cells of flow biofilms were visualized under a microscope. Indeed, orf19.2500−/− hyphae showed a significant reduction in lateral yeast growth compared to WT biofilm hyphae (**Fig 1E**, **S1B Fig**), clarifying the reason for decrease in biofilm dispersal in the mutant.

### Orf19.2500 −/− has a wild-type growth rate and morphology in glucose, but fails to grow on alternative carbon sources or in the presence of stressors

**Fig 2:**
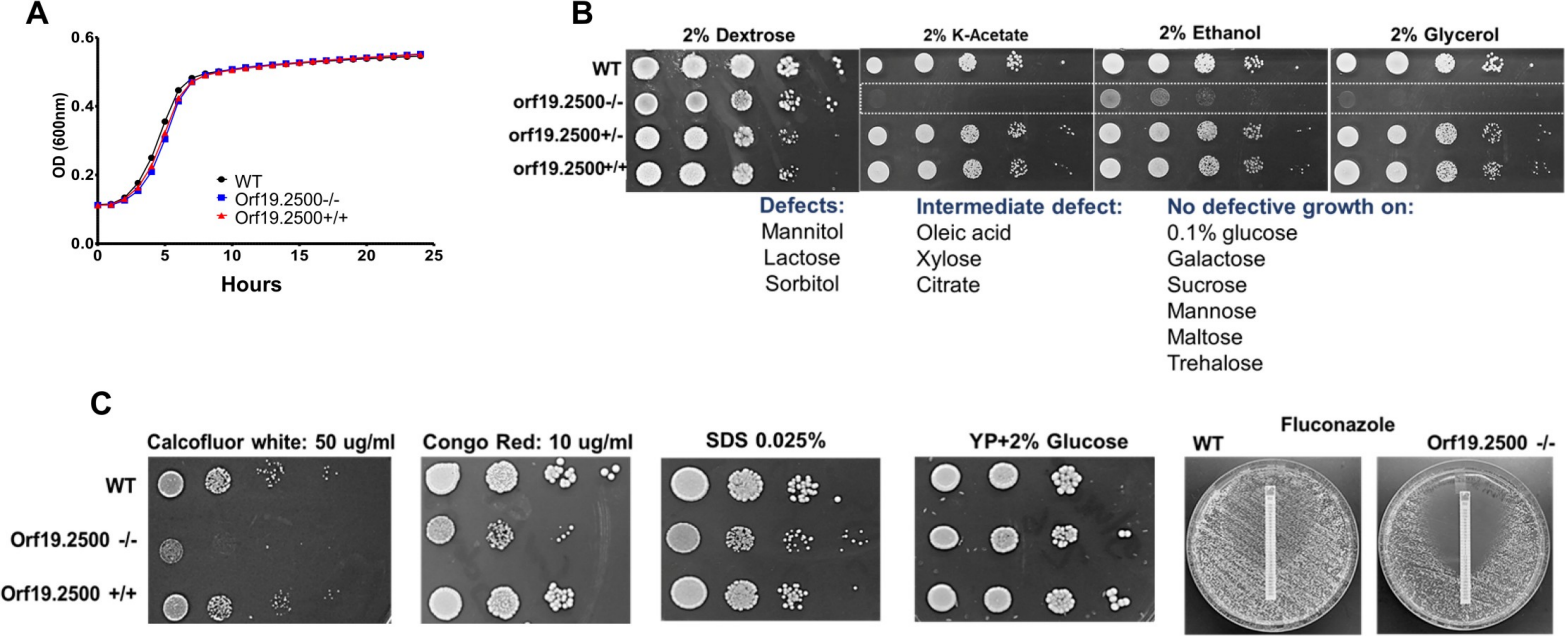
Pattern of growth on various carbon sources and stressors. The orf19.2500−/−, WT, and revertant strain were grown in 10% YP+2% glucose media, and OD600 of the growth was measured temporally, using a spectrophotometer (A). Different concentrations (5 μl of 10^5^ cells/ml to 10^1^ cells/ml) of WT, orf19.2500 mutant −/−, heterozygote +/−, and revertant strain +/+ were spotted on solid YP media containing 2% of glucose, various alternative carbon sources, or media containing cell wall/membrane stressors. Differences in extent of growth were visually noted (B, C). Raw data are found in the file S1 Data. WT, wild-type.

The fact that orf19.2500−/− was able to make robust biofilms indicated that it may not be deficient in growth or morphogenesis. We performed assays to measure the growth of the mutant in comparison to WT and complemented strains, under planktonic conditions. We found that in the first 24 h, the growth curves of orf19.2500−/− exactly overlapped with the other 2 strains, when grown in rich media containing 2% glucose (**Fig 2A**, raw data are found in the file S1 Data). In fact, the mutant and the WT controls displayed comparable growth rate and viability until day 4, after which the mutant cells gradually lost viability at significantly higher rates than the WT cells (**S1C Fig**, raw data are found in the file **S1 Data**). Visual examination of colonies grown on solid agar media from 4-day-old cultures showed that the mutant cells were significantly smaller in size compared to WT cells, pointing to a defect in respiratory capacity post glucose exhaustion (**S1D Fig**). Microscopic visualization and measurement of hyphal lengths revealed no significant differences in hyphal growth and elongation between WT and mutant cells and correspondingly no defect in their capacity to damage human vascular endothelial cells (**S1E and S1F Fig**, raw data are found in the file S1 Data).

It is well known that growth of petite sized colony of mutants is limited to fermentable carbon sources [10]. As such, orf19.2500−/− grew robustly on media with glucose, but displayed severe growth defects on alternative carbon sources such as acetate, ethanol, and glycerol (**Fig 2B**). The WT, heterozygote, or the complemented strain did not exhibit this defect. This growth arrest in a non-fermentable carbon source could be reversed by addition of glucose into the acetate media (both liquid and solid growth conditions), indicating the orf19.2500 mutant required glucose for growth. As shown in **S1G Fig left panel**, growth of mutant in 1% acetate containing YP broth was negligible after 24 h, compared to WT and revertant strains. Addition of 1% glucose into these tubes regenerated growth overnight in the mutant strains. In fact, this was also tested on solid media. We spread of lawn of WT or mutant strain on 1% acetate medium, incubated the plates for 4 h, and then placed a sterile filter paper disc containing 2% glucose on the plates. After 24 h of incubation, while the WT cells grew all over the acetate plate, regrowth of orf19.2500 mutant was seen in the form of small colonies only around the glucose disc, indicating that glucose was indeed important for orf19.2500 mutant growth (**S1G Fig**, 2 right panels).

We probed the extent of growth deficit of the mutant further, in the presence of cell wall and cell membrane stressors. Compared to the WT or the orf19.2500+/+ complemented strains, orf19.2500−/− disruption mutant was significantly more sensitive to growth on calcofluor white, congo red (cell wall stress), and SDS or fluconazole (cell membrane stress) (**Fig 2C**). In fact, on examination by staining with concanavalin A (stains cell surface mannans) or calcofluor white (stains cell wall chitin) followed by flow cytometry, the mutant strain displayed at least 40% to 50% reduced cell surface mannan or chitin content compared to the WT or complemented cells (**S2A Fig**). This deficiency was further corroborated by transmission electron microscopy, which displayed a strikingly thinner mannan layer, and at least 35% decrease in overall cell wall thickness in mutant cells versus the WT, as quantified by measuring the cell wall thickness of multiple cells, at various sections (**S2B Fig**, raw data are found in the file S1 Data).

To understand why the mutants were susceptible to cell membrane stressors, we investigated the membrane permeability of WT and mutant cells, as a measure of membrane integrity. Orf19.2500−/− mutant was significantly more permeable to fluorescein diacetate (FDA, a membrane intercalating dye), while WT cells completely blocked the membrane penetration of FDA, indicating that the cell membrane of the WT was healthier than that of the mutant (**S2C Fig**, raw data are found in the file S1 Data). As expected, the membrane disrupting antifungal drug fluconazole, which was used as a positive control, showed enhanced uptake of FDA in both WT and mutant cells. Since ergosterol is a major component of the *C*. *albicans* cell membrane [11], we questioned if ergosterol production was impaired in the mutant. Gene expression analysis of select *ERG* genes indicated that the orf19.2500−/− had a 2.7-fold increased expression of *ERG20*, which is a putative farnesyl pyrophosphate synthase, required for both coenzyme Q and ergosterol biosynthesis. Most other *ERG* genes downstream of *ERG20*, solely important for ergosterol biosynthesis, were down-regulated >2- to 3-fold (**S2D Fig**, raw data are found in the file S1 Data), perhaps signifying the reason for higher membrane permeability and fluconazole susceptibility in the mutant.

### Orf19.2500 localizes to the mitochondria and plays a key role in functioning of Complex I of the mitochondrial electron transport chain

**Fig 3:**
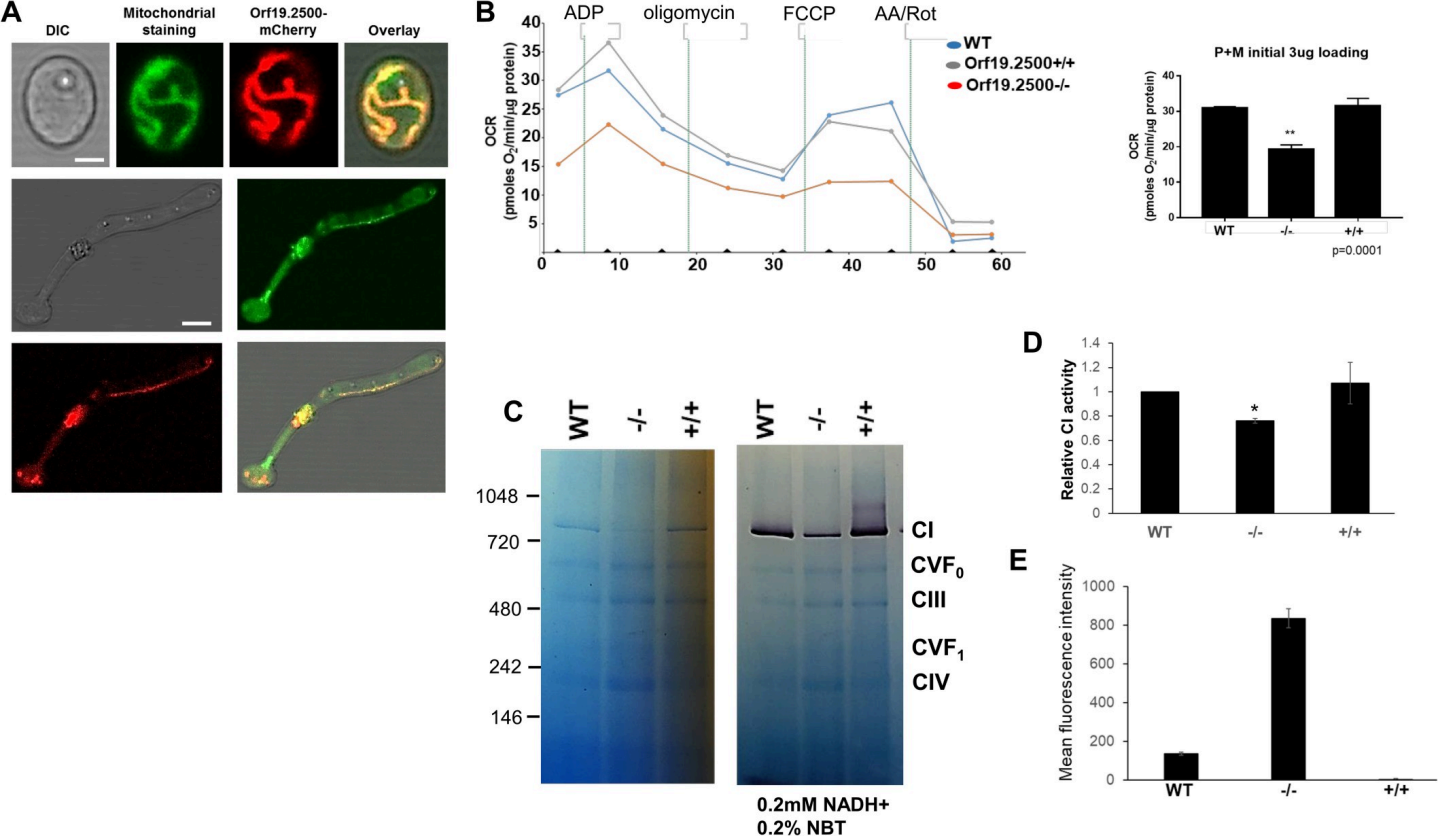
Organelle localization of orf19.2500 and measurement of its defect in respiration and mitochondrial complex stability. Orf19.2500 was engineered under a constitutively expressing tet-promoter, tagged with mCherry. Localization of the orf19.2500 was determined by staining both yeast and hyphal cells with a green fluorescent mitochondrial stain (Mitotracker green) to display the yellow overlap of colors in the mitochondria. Scale bar for top panel is 2 μM, bottom panels 5 μM. (A). The XF96 Analyzer was used to measure changes in mitochondrial bioenergetics by measuring the OCR in freshly isolated mitochondria of WT, mutant, and revertant strains. One μg each of ADP, oligomycin, FCCP, and antimycin A and rotenone were added at the indicated points. The maximal respiratory capacity was quantified (B). Values are means ± SEM. **(*p* < 0.01 mutant vs. WT and revertant; measurements from 6 independent isolations). BN-PAGE electrophoresis of equal quantities of total mitochondrial proteins of *C*. *albicans* WT, orf19.2500−/−, and orf19.2500+/+ cells, stained with Coomassie to reveal respiratory complexes CI to CIV (C). Raw data are found in the file S1 Raw Images. The molecular markers indicated to the left are NativeMark (unstained protein standard; Invitrogen). The in-gel enzyme activity of CI in BN-PAGE was assayed within 60 min after incubating the gel in reaction medium (0.1 M Tris-HCL, pH 7.4, 0.2 mM NADH as a substrate, and 0.2% NBT). Specific activity of CI in mitochondria from mutant and complemented strains were quantified and plotted relative to the CI activity in WT mitochondria (* = *p* < 0.05) (D). ROS activity in WT, mutant, and complemented strains were measured by staining the cells with MitoSox red, and measuring fluorescence intensity was measured by flow cytometry and plotted (E). Raw data are found in the file S1 Data. BN-PAGE, Blue native PAGE; CI, Complex I; CIV, Complex IV; OCR, oxygen consumption rate; ROS, reactive oxygen species; WT, wild-type.

In a quest to understand the function of this protein, we endeavored to unravel its cellular location. Attempts to tag 1 allele of orf19.2500 with a fluorescence tag were not productive due to a faint fluorescence signal, which, although visible under the microscope, could not produce clear images for documentation. Because the expression levels of orf19.2500 were low, we constructed a tetracycline regulatable strain, which also harbored an mCherry marker right after the Tet-off promoter (orf19.2500/*ORF19*.*2500*-mCherry-tetO). In rich media containing glucose, there was an overexpression of orf19.2500-mCherry and the protein in the cell fluoresced red. The red fluorescence completely overlapped with a stain that colors the mitochondrial matrix green to provide an overall yellow colored mitochondrial localization (**Fig 3A**). Thus, the protein localized to the mitochondria in both yeast and hyphae.

The fact that orf19.2500 mutant could not grow on alternative carbon sources indicated a respiratory defect likely due to a faulty ETC [12]. To test this hypothesis, we carried out Seahorse assays to test the respiratory prowess of the isolated mitochondria of the mutant strain, in the presence of CI substrates (pyruvate + malate) (**Fig 3B**, raw data are found in the file S1 Data). Compared to the mitochondria from the WT or the complemented strain, the orf19.2500−/− mutant mitochondria showed an overall significant decrease in respiratory capacity, measured by a 30% decrease in oxygen consumption rate (OCR). This was not the case when a Complex II (CII) substrate (succinate in presence of rotenone) was used; mitochondria from all 3 strains displayed equivalently robust CII activity (**S3A Fig**, raw data are found in the file S1 Data).

To further test if CI was impacted in the mutant strain, we performed a Blue native PAGE (BN-PAGE) analysis, in which the 5 different complexes of the ETC from isolated mitochondria (of each strain, respectively) were separated by electrophoresis and CI activity tested. We determined that CI is reduced by 40% to 50% in orf19.2500−/− compared to the gel density ratios of CI/CIII and CI/CV to WT or orf19.2500+/+ cells by ImageJ (**Fig 3C** left panel; mean band intensities WT: 3,777+50, mutant: 2,184+32, revertant: 4,914+79; *p* < 0.01 mutant versus WT or rev). In addition, the in situ assay of CI NADH dehydrogenase enzyme activity demonstrated that mutant strain correspondingly had reduced enzyme activity than the WT or reconstituted mitochondria (**Fig 3C** right panel; Image J mean band intensities WT: 34,382+503, mutant: 14,403+139, revertant: 37,799+66; *p* < 0.01 mutant versus WT or rev). Quantitative measurement of enzymatic activity independently confirmed an approximately 30% decrease in CI in the mutant compared to the WT strains (*p* < 0.05) (**Fig 3D**, raw data are found in the file S1 Data). Thus, the Seahorse assays (**Fig 3B**) reduced assembled CI and its enzymatic activity (**Fig 3C and 3D**), supporting the hypothesis that orf19.2500 is important for CI activity in *C*. *albicans*. Based on its role in mitochondrial oxidative phosphorylation and its NADH dehydrogenase activity, Orf19.2500 was renamed as *NDU1* for NADH dehydrogenase of CI.

Since CI is the major donor to the proton gradient [13], we posited that a reduction in its activity would affect the mitochondrial membrane potential (ΔψΜ). WT, mutant, and complemented strains were grown overnight in YP+2% glucose and then subcultured in media containing either glucose or acetate as a carbon source. After 2 h of growth, cells were treated with JC1, a dye used as an indicator of mitochondrial membrane potential. Compared to WT and complemented strains, *NDU1* mutant cells had significantly higher mitochondrial membrane depolarization, as measured by the intercalation of JC1 dye, and the shift in the green FITC fluorescence. However, this reduction in membrane potential was found only under nutritional stress, such as in the presence of the alternative carbon sources of acetate (**S3B Fig**), and not during growth on glucose.

CI is responsible for most cellular reactive oxygen species (ROS) production in mitochondria [13], and impairment in CI activity often results in oxidative stress. The accumulation of mitochondrial ROS was determined by measuring the superoxide levels with MitoSOX Red dye, which is specifically targeted to mitochondria in live cells. Oxidation of MitoSOX Red reagent by superoxide produces red fluorescence, which is quantified by flow cytometric analysis and correlated with the amount of ROS present in the mitochondria. *NDU1* deletion led to an elevation in the mitochondrial superoxide levels, which, upon quantification of flow cytometric data, revealed a greater than 6-fold increase in MitoSOX staining in *NDU1* mutant cells versus the WT or complemented strains (**Fig 3E**, raw data are found in the file **S1 Data**).

### *NDU1* is hypersusceptible to neutrophil killing and avirulent in a mouse model of hematogenously disseminated candidiasis

**Fig 4:**
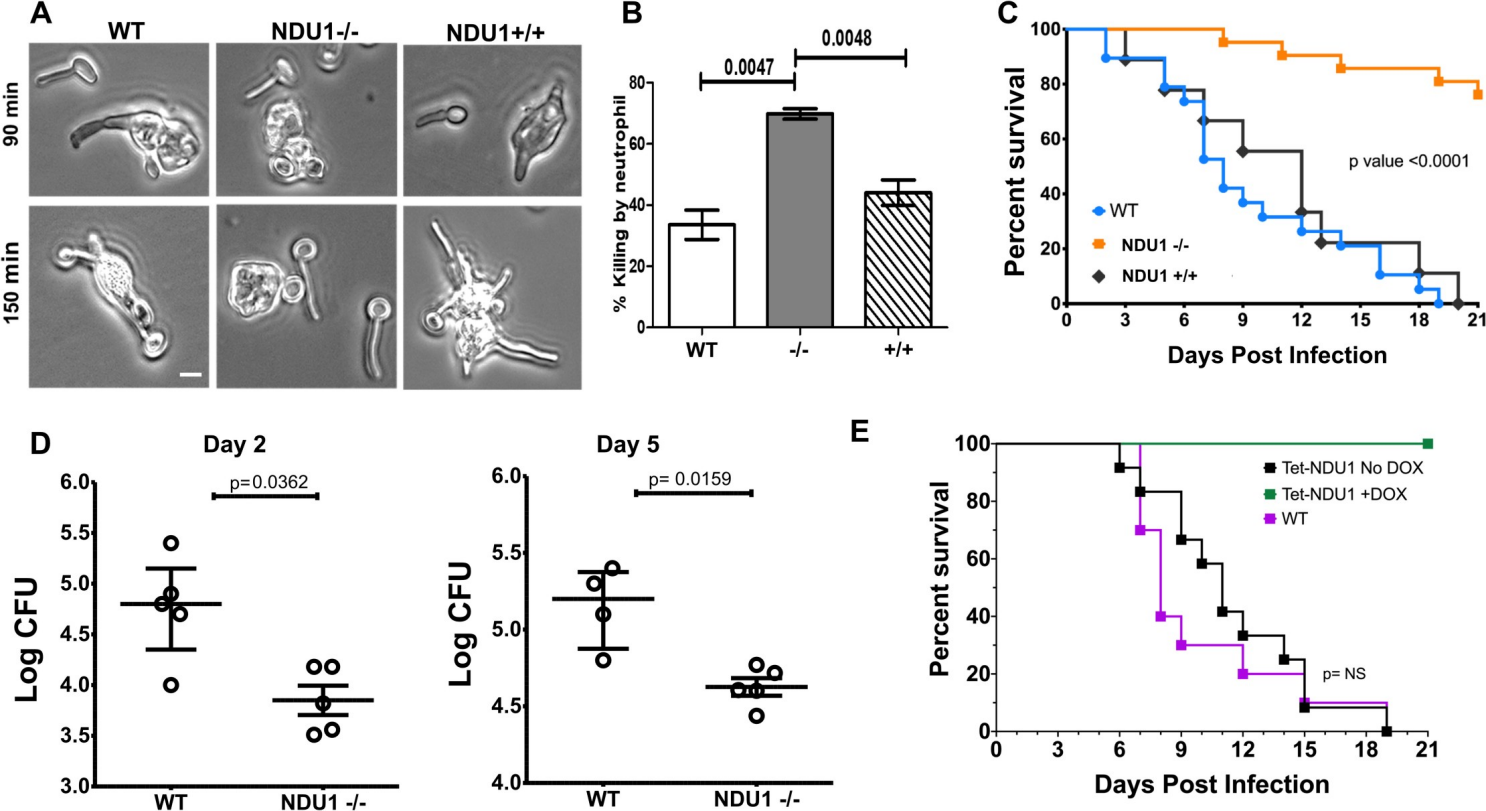
Susceptibility to neutrophils and defective virulence of NDU1 mutant. (Note: orf19.2500 is referred as NDU1 from this figure on). Yeast *C*. *albicans* WT, NDU1−/−, and NDU1+/+ cells grown overnight were incubated along with primary human neutrophils (3:1 MOI), for 3 hours, and the extent of phagocytosis was visualized by confocal microscopy at 90 min and 150 min (A). The extent of yeast cell killing by neutrophils was quantified by CFU measurement at 3 h. Scale bar = 5 μM (A). Data are mean + SEM from 3 biological replicates. CD1 outbred mice were infected via tail vein with 2.5 × 10^5^ cells of WT, NDU1−/−, and NDU1+/+ (10 mice each × 2 replicates), and the impact of disseminated candidiasis on the overall survival of mice was monitored for 21 days. *p < 0.0001 mutant vs. WT and revertant, log-rank test (B). Mice were sacrificed on day +2 and +5 relative to infection, and their kidneys processed for tissue fungal burden, determined by plating on solid media. Data are median ± interquartile range. *p < 0.05 mutant versus WT, Wilcoxon rank-sum test (C). Survival curves for the different groups of mice (10 animals per group) infected with the *C*. *albicans* WT isogenic parental strain or with the *C*. *albicans* tetO-*NDU1*/*ndu1* strain in the presence or absence of DOX. Statistically significant differences were measured between the comparison groups of mice (D). Survival curves for the different groups of mice (10 animals per group) infected with the *C*. *albicans NDU1/NDU1* isogenic parental strain (WT) or with the *C*. *albicans* tetO-*NDU1/ndu1* (Tet-NDU1) strain in both the presence and the absence of DOX. DOX was added in the water 24 h after *C*. *albicans* infection. Statistically significant differences were observed between the comparison groups of mice Tet-NDU1+DOX versus WT or Tet-NDU1 No DOX (*p* < 0.001). Survival between the latter 2 groups was not significantly different (NS; *p* = 0.8). Raw data are found in the file S1 Data. CFU, colony-forming unit; DOX, doxycycline; WT, wild type.

Considering that *NDU1* mutants are unable to grow on alternative carbon sources, we hypothesized that they would have difficulty surviving in the nutritionally deprived environment of innate immune cells [14]. To test this, we determined the killing ability of these mutant strains by primary human neutrophils. Within 45 min, neutrophils had engulfed yeast cells of all 3 strains. By 90 to 150 min, *C*. *albicans* WT as well as the complemented strain developed germ tubes and were able to destroy the immune cells (**Fig 4A**). In contrast, the *NDU1* null mutant remained as engulfed yeast cells inside the neutrophils, and by 3 h, were eventually killed in significantly (2-fold) higher numbers than WT or *NDU1* complemented strains (**Fig 4B**, raw data are found in the file S1 Data).

The inability of *NDU1*−/− to evade the immune system translated predictably into avirulence in a hematogenously disseminated mouse model. While 100% of the mice succumbed to infection by the WT or complemented strains within 21 days, >80% of the mice infected with *NDU1*−/− null mutant survived the infection (**Fig 4C**). This striking defect in survival was corroborated with approximately 0.5 to 1.0 log reduction in kidney fungal burden of mice infected with the *NDU1*−/− versus those harvested from mice infected with the WT and collected 2 or 5 days postinfection (**Fig 4D**).

We also studied the virulence of the generated mutant strain at 10-fold higher infectious dose of 2.5 × 10^6^ cells. Interestingly, while mice infected with WT and complemented strains succumbed early to the infection within 7 days, approximately 80% of mice infected with the *NDU1*−/− mutant survived the infection (**S3C Fig**, raw data are found in the file **S1 Data**), thereby mimicking the survival of mice infected with the lower infectious dose (**Fig 4C**, raw data are found in the file **S1 Data**).

The fact that the mutant strain did not cause virulence could likely be attributed to their early susceptibility to phagocytes or their defective long-term sustenance in a glucose-impoverished environment in vivo. To test this further, we constructed a *C*. *albicans* strain, wherein 1 allele of *NDU1* was deleted while the other was placed under a tetracycline-regulatable promotor (*NDU1Δ*/*NDU1*-tetO). The expression of *NDU1* could be increased or decreased based on the presence or absence of doxycycline (DOX) in the growth milieu. For virulence studies, 1 set of mice was infected via tail vein with WT, and 2 other sets with the *NDU1*-tetO strain. Mice were fed with plain water for 24 h after infection, to enable unrestrained dissemination, after which DOX was added (at 24 h after infection) to the drinking water of 1 set of *NDU1*-tetO–infected mice (to deplete expression of *NDU1*) and to the set infected with WT (DOX control). The third set of mice were fed continuously with water without DOX (for overexpression of *NDU1*). As clearly seen in **Fig 4E**, sustained depletion of *NDU1* in vivo due to DOX in systemic circulation translated into 100% mouse survival rate, while the WT and overexpression strains demonstrated similar levels of lethality in mice.

### *NDU1* 3D structure has characteristics of a dehydrosqualene synthase and is homologous to human NDUFAF6

**Fig 5:**
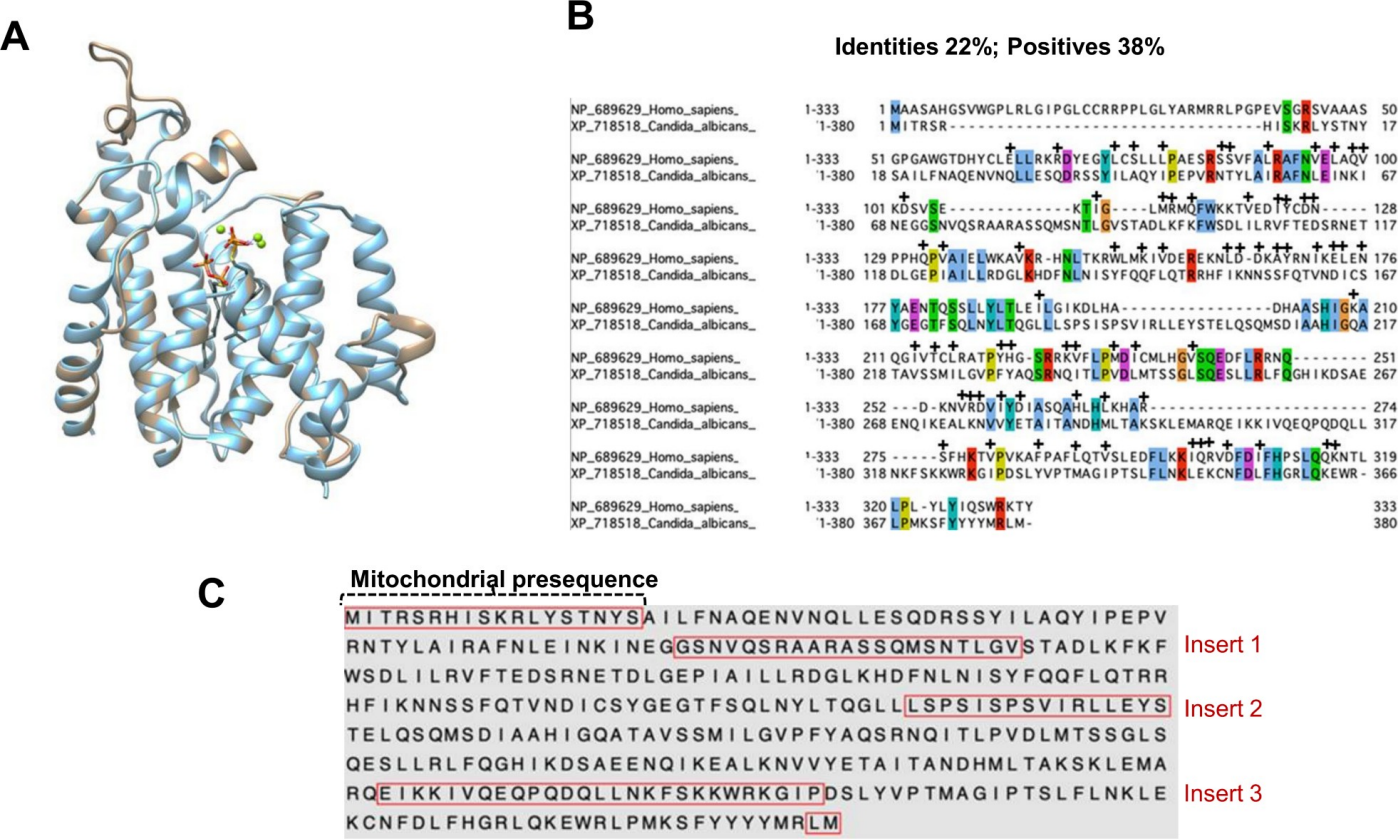
Overlay of template and NDU1 models The Phyre2 model of NDU1, c5iysA (blue), is overlaid onto the structure 5iys (tan) with an RMSD of 0.270 Å between 253 atom pairs. Two molecules of the substrate analog, FPS and 3 Mg^++^ ions (green), are bound in the 5iys active site cavity. (A). BLAST alignment of *C*. *albicans* NDU1 with human NDUFAF6. Colored amino acids represent identity, while + indicates positives (B). Protein sequence of NDU1 with the highlighted mitochondrial presequence, and 3 inserts acquired over evolution, which sets *C*. *albicans* NDU1 apart from its human orthologue (C). FPS, farnesyl thiopyrophosphate.

We predicted that the key to identifying NDU1 protein function lay in unraveling its three-dimensional (3D) structure. The NDU1 sequence was submitted to MitoProt II for analysis [15]; a mitochondrial targeting sequence of 15 amino acids was predicted to be removed with high probability (0.9124), suggesting that the mature NDU1 (mNDU1) protein begins at Asn16. Structural models for NDU1 were generated by submitting the mNDU1 protein sequence to Phyre2 (V 2.0), a protein homology recognition engine that uses profile–profile matching algorithms [16]. A model for NDU1 named c5iysA was generated with 100% confidence by threading the NDU1 sequence onto chain A of 5IYS. 5IYS is the crystal structure of the *Enterococcus hirae* dehydrosqualene synthase in complex with 2 molecules of the substrate analog, farnesyl thiopyrophosphate (FPS) (**Fig 5A**). An overlay of c5iysA and 5iys has an RMSD of 0.270 Å between 253 pruned atom pairs, an excellent match, especially over the core regions. The 2 molecules of FPS are bound in a large “pocket” (2087 Å^3^) in 5iys (**S4A Fig**). The c5iysA model (NDU1 threaded onto 5iys) also has a large pocket as determined by Castp [17] (2332 Å^3^) (**S4B Fig**). The pocket is larger than that in 5IYS but has a somewhat different shape and cannot accommodate the FPS molecules exactly as positioned in 5IYS. Nonetheless, the FPS lipid chains are highly flexible and can likely adapt to the c5iysA pocket. The second model predicted by Phyre2 was c4hd1A, which is modeled on 4hd1 which is a squalene synthase from *Alicyclobacillus acidocaldarius* (**S4C Fig**). Overlay of c4hd1A (green) and 4hd1 (purple) yielded an excellent RMSD between 245 pruned atom pairs as 0.262 angstroms. Thus, NDU1 is a predicted squalene/phytoene synthase (pfam: PF00494).

We also unraveled that *C*. *albicans* NDU1 has a human orthologue NDUFAF6, the NADH:ubiquinone oxidoreductase complex assembly factor 6, which shares approximately 22% identity (38% similarity) (**Fig 5B**, raw data are found in the file S1 Data). Location of specific NDU1 residues (red) with identities to NDUFAF6 (gray) is displayed in a structural schematic in (**S4D Fig**). NDUFAF6 is considered a member of the Isoprenoid_Biosyn_CI superfamily, which generates tens of thousands of isoprenoid metabolites, including sterols, heme, dolichol, carotenoids, and ubiquinones.

### *C*. *albicans* NDU1 has evolutionarily acquired amino acid inserts unique to CTG clade fungi

**Fig 6:**
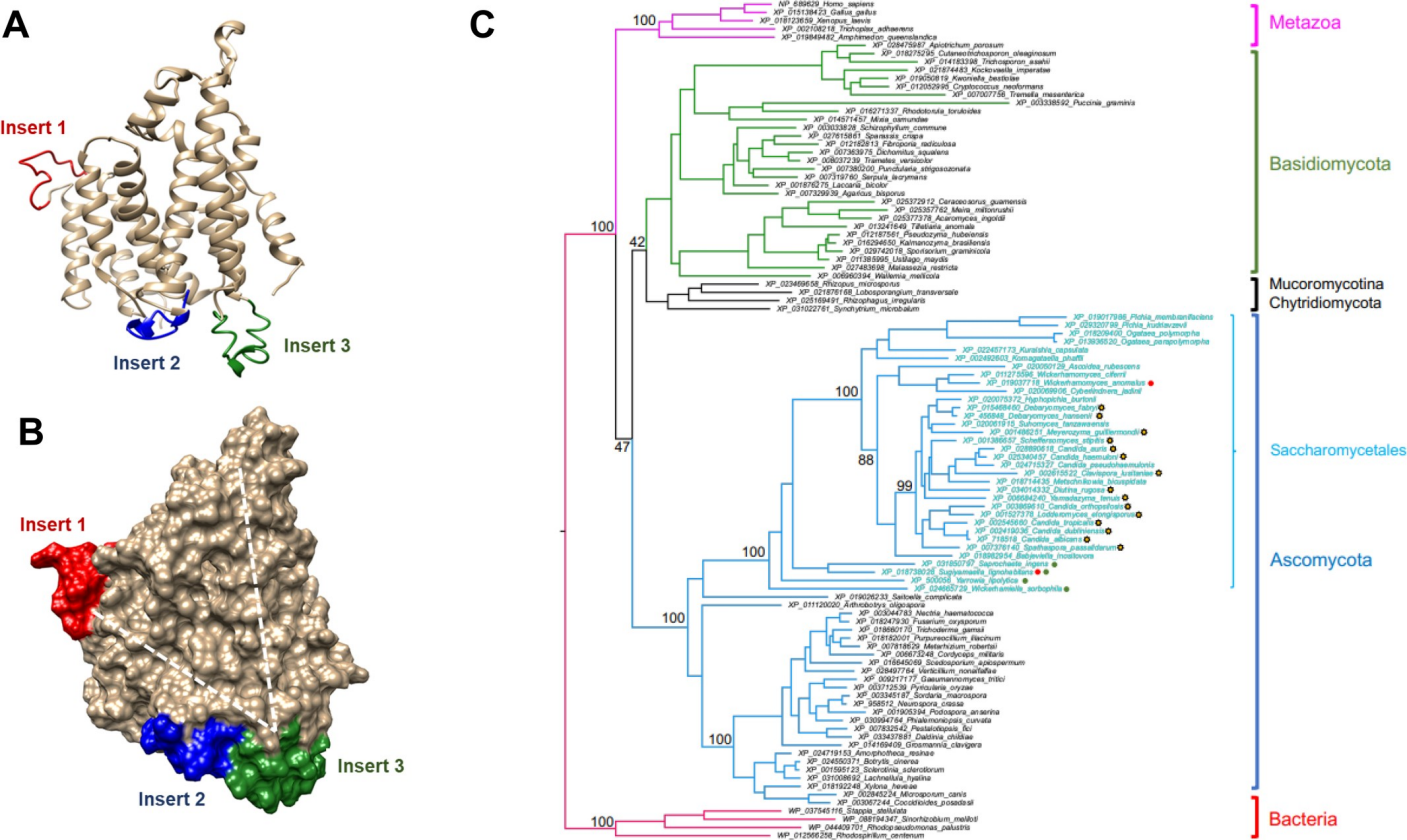
Location of the 3 insertion sequences of NDU1 and their phylogenetic uniqueness. The unique insertion sequences of NDU1 were localized on ribbon and on surface representations of the iTASSER model (A). Note how insert 2 (blue) and 3 (green) lie to the bottom of the V-shaped cavity highlighted by dotted lines (B). A maximum likelihood tree of selected eukaryotic and prokaryotic NDU1 orthologs emphasizes the highly restricted distribution of the 3 insert sequences. This tree has 4 bacterial (red), 5 metazoan (magenta), and 93 fungal orthologs, with branches colored green for Basidiomycota, black for Chytridiomycota and Mucoromycotina, and blue for Ascomycota. Tips belonging to the order *Saccharomycetales* are colored cyan. Bootstrap support values for selected nodes are given. Proteins belonging to the CTG clade of yeasts are noted with a yellow star. All *Saccharomycetales* proteins have insert 2; 2 are completely missing insert 1 (red circles); 4 are completely missing insert 3 (green circles) (C).

Protein sequence alignments and 3D homology modeling between NDU1 and NDUFAF6 further revealed that NDU1 is a longer protein (380 versus 333 amino acids of NDUFAF6), and it has extra stretches of amino acid inserts that are depicted as gaps in the human NDUFAF6 sequence (**Fig 5B**). Specifically, NDU1 has 4 prominent amino acid inserts that are evolutionarily acquired within its protein sequence (**Fig 5C**). The part of sequence highlighted at the N-terminus is the predicted mitochondrial targeting sequence, which is truncated upon import to the mitochondria, hence is irrelevant to function. The red-boxed inserts 1, 2, and 3 are unique to NDU1. These inserts are not modeled in the c5iysA model from PHYRE2, as they are not present in the 5IYS template. The mNDU1 sequence was submitted for modeling to iTASSER, which employs threading template identification and iterative modeling to model the entire sequence [18]. On the iTASSER model, the 3 inserts lie in loops on the surface of the protein model (**Fig 6A**). In fact, when visualized on a surface model, insert 1 is located at the opening of the NDU1 pocket. The pocket/cavity is formed by long alpha helices packing together, and inserts 2 and 3 were modeled to the bottom of the V-shaped pocket cavity, between those alpha helices (**Fig 6B**). Additionally, the latter 2 inserts were found close together in 3D space, enough to be in contact with each other.

To investigate the phylogenetic distribution of the insertion sequences in orthologous proteins, we performed a Psiblast search using *C*. *albicans* NDU1 (XP_718518) as query against the RefSeq (release 200; 2020/05/04) database. Putative bacterial and eukaryotic orthologs of NDU1 with evalues < 1e-30 were aligned with MAFFT [19], a multiple sequence alignment software, and a phylogenetic tree was generated using IQ-TREE [20]. The tree was used to manually reduce the number of sequences outside the *Saccharomycetales*, while retaining phylogenetic diversity. The final tree contains 102 putative NDU1 orthologs and uses the bacterial orthologs as an outgroup: 4 bacterial (red), 5 metazoan (magenta), and 93 fungal orthologs, with branches colored green for Basidiomycota, black for Chytridiomycota and Mucoromycotina, and blue for Ascomycota. Only the Saccharomycetales (in particular the CTG clade yeasts noted with a yellow star) were found to contain all 3 inserts. This group had longer branches and were well separated from the other groups, indicating greater divergence in the NDU1 sequences over evolution. Interestingly *Saccharomyces cerevisiae* and *Candida glabrata* are not included in the tree because they do not have CI, and hence lack *NDU1* orthologues (**Fig 6C**).

To elaborate further, based on the presence or absence of the 3 insertion sequences, the phylogenetic tree was clearly divided into 3 groups (**S4E Fig**): group 1 colored blue contained Saccharomycetales and *Candida* like CTG clade fungi (CTG clade), group 2 in black which had other fungi, and group 3 colored pink represented sequences from bacteria and eukaryotes. Group 1 had longer branches and were well separated from group 2 and 3 sequences. Also, only group 1 had all 3 insert sequences. In contrast, group 2 had no insert 1 or insert 2 and had a different insert 3, while group 3 were lacking in all the inserts (**S4E Fig**). Overall, our analyses showed that the *C*. *albicans* mitochondrial protein NDU1 has structures distinct to CTG clade proteins, and these inserts may be functionally important for enzymatic activity or protein–protein interactions, distinct for *Candida* spp.

### Expression of the human NDUFAF6 does not complement *C*. *albicans* NDU1 defect, while insert 2 and 3 are the functional hub of NDU1

**Fig 7:**
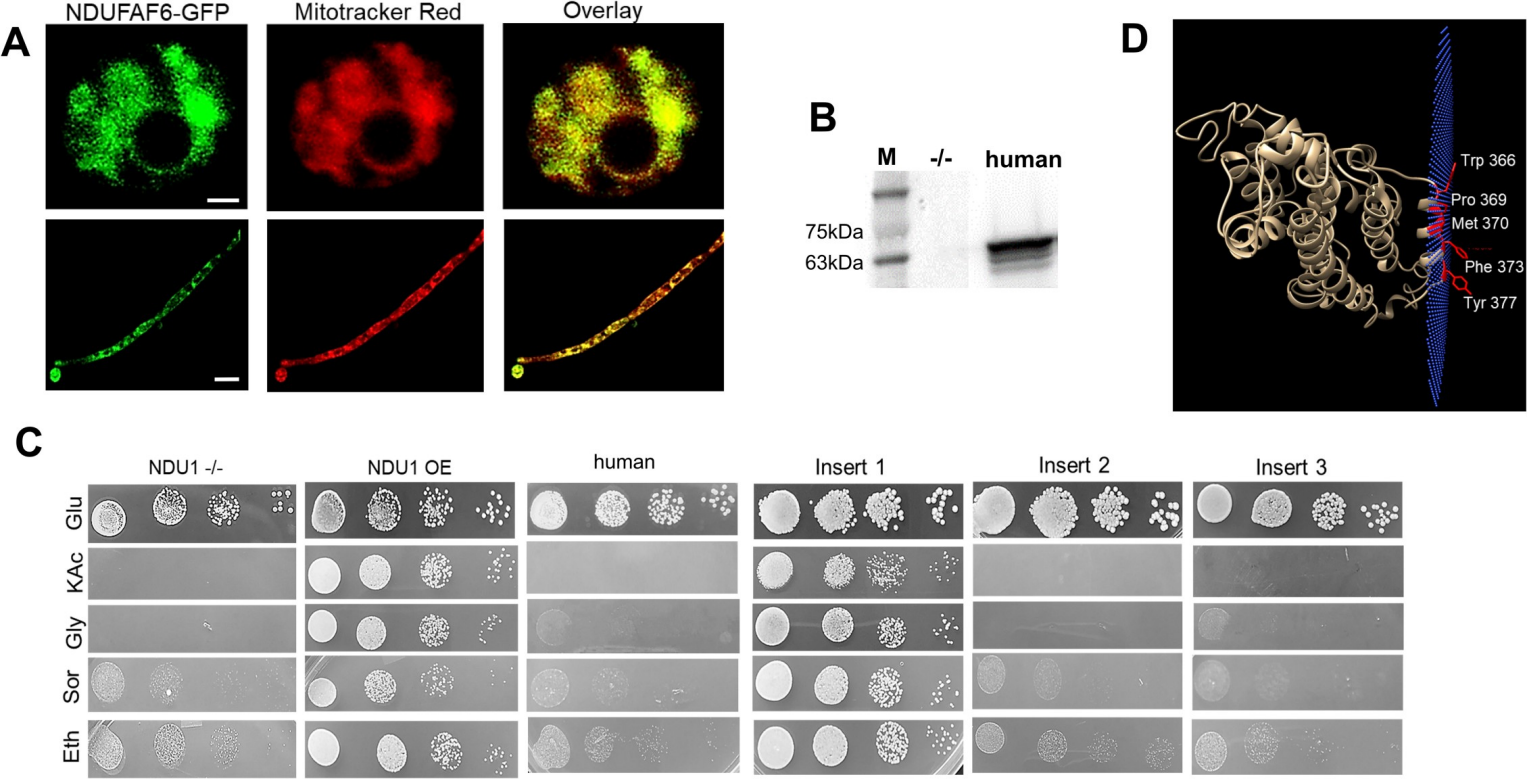
Expression and localization of human NDUFAF6 in *C*. *albicans*. Entire ORF of GFP-tagged human NDUFAF6 was expressed in *C*. *albicans* NDU1 mutant and found to be localized to mitochondria, as visualized by GFP overlapping with a mitochondrial stain, in both yeast and hyphae. Scale bar for top panel 1 μM, bottom panel 10 μM (A). Detection of the GFP-tagged human NDUFAF6 protein by western blotting using an anti-GFP antibody. M = marker, second lane is total protein from *NDU1* mutant (negative control), lane 3 is the 63-kDa human NDUFAF6-GFP fusion protein detected in total protein of NDU1 mutant strain expressing the human NDUFAF6 (B) Raw data are found in the file S1 Raw Images. *C*. *albicans* NDU1 mutant expressing NDUFAF6 grows on glucose but not on 2% acetate (B). Individually expressed entire ORF of NDU1 (NDU1 overexpression strain), or NDU1 lacking each insert, in the NDU1 mutant, were screened for their ability to grow on alternative carbon sources. The NDU1 mutant was used as the parent control strain for the experiment (C). OPM server predicts NDU1 is a peripheral membrane protein (plane of blue spheres) and identifies 5 membrane-embedded amino acids (D).

Since NDU1 was important for immune evasion, drug resistance, and virulence in *C*. *albicans*, we questioned if it would represent a viable target for antifungal drug development. Specifically, we probed the functional diversity of NDU1 compared to its human counterpart NDUFAF6. Hence, we first inspected if heterologous expression of human NDUFAF6 in *C*. *albicans* could provide a gain of function in the NDU1 mutant strain. The entire codon-optimized ORF of NDUFAF6 was expressed constitutively under a tet-regulatable promoter (codon was optimized by Genscript, NJ, for expression in CTG clade yeast). Importantly, the expressed protein also harbored a GFP-tag. NDUFAF6-GFP localized correctly to the mitochondria of both yeast and hyphae (**Fig 7A**), and western blotting of *C*. *albicans* total protein with anti-GFP antibodies yielded the correct size NDUFAF6 protein (approximately 63 kDa; NDUFAF6 38kDa+GFP 25 kDa), in the *NDU1* mutant expressing the human protein (lane 3; **Fig 7B**). As expected, no band was detected by the anti-GFP antibodies in the negative control represented by total protein from the *NDU1* mutant (lane 2; **Fig 7B**). We tested if expression of the human orthologue could revert *C*. *albicans* NDU1 mutant growth defect on acetate. As expected, the control *NDU*−/− mutant did not grow on acetate, glycerol, sorbitol, or ethanol, while strains overexpressing the full-length *C*. *albicans NDU1* (tet-regulatable NDU1; OE) grew robustly on the non-fermentable carbon containing media (**Fig 7C**). We also found that human NDUFAF6 expression did not restore growth of the mutant on 2% potassium acetate or other alternative carbon sources such as ethanol, glycerol, or sorbitol (**Fig 7C**), indicating that the human protein could not revert the functional defect of NDU1 on non-fermentable carbon sources.

To validate this further, we probed if the 3 insert sequences unique to *C*. *albicans* NDU1 had a role to play in mitochondrial function determined by growth on non-fermentable carbon sources. As done with the human protein, we expressed the entire ORF of *C*. *albicans* NDU1 minus the individual inserts, in the *C*. *albicans NDU1* mutant. Similar to the *C*. *albicans* mutant expressing the human protein, mutants expressing the mutated *C*. *albicans* NDU1 constructs expressed proteins that localized to the mitochondria, and were expressed at the right size, as evaluated by fluorescence microscopy (**S5A Fig**) and western blots (**S5B Fig**), respectively. The band sizes on western blots were as expected, NDU1 minus insert 1 + GFP = 65.1 kDa; NDU1 minus insert 2 + GFP = 64.7 kDa; NDU1 minus insert 3 + GFP = 63.5 kDa). Interestingly, we found that strains expressing NDU1 with a truncated insert 1 grew as good as the OE strain, indicating that this area was dispensable for protein function. In contrast, mutants with individually truncated insert 2 or 3 maintained the growth defect on alternative carbon sources, highlighting their importance to the function of NDU1 (**Fig 7C**). All strains grew robustly on glucose containing media.

### NDU1 is a peripheral membrane protein

Isoprenoid substrates and products are hydrophobic and are predicted to be localized to the membrane. NDU1 was not found to have predicted transmembrane segments (using transmembrane prediction servers such as TMHMM Server v. 2.0 or PRED-TMR of the SwissProt database). We then submitted the iTASSER model to the Orientations of Proteins in Membranes (OPM) server, which determines the spatial position of membrane boundaries for integral and peripheral membrane proteins [21]. OPM optimizes the free energy of protein transfer (ΔG_*transf*_) from water to a membrane environment. For peripheral membrane proteins, ΔG_*transf*_ varies between −15 and −1.5 kcal mol^−1^. OPM strongly suggested that NDU1 is a peripheral membrane protein with optimal energy of −7.6 kcal/mol and 5 residues of the carboxyl-terminal helix (Trp356, Pro369, Met370, Phe373, and Tyr377) were predicted to be membrane embedded (**Fig 7D**). The model also predicted the NDU1 cavity to lie above the mitochondrial membrane (**S5C Fig**).

## Discussion

Biofilm formation and dispersal are determinants of virulence in *C*. *albicans*. We used genetic, bioinformatic, and biochemical approaches to identify *NDU1*, a gene that encodes a mitochondrial protein that is evolutionarily divergent from other eukaryotic orthologues. *NDU1* affects biofilm dispersal and detachment, helps resist phagocyte killing, and is important for full virulence of *C*. *albicans* in mice. The most compelling phenotype of *NDU1* null mutant is its inability to grow on non-fermentable carbon sources or in advanced stationary growth when glucose is depleted. In *S*. *cerevisiae* and *C*. *albicans*, respiration-deficient mitochondrial mutants are unable to grow on non-fermentable carbon sources [22] and form petite colonies because their cell division rates are lower than that of the normal cells [23]. Consistent with these observations, and as predicted by the N-terminus mitochondrial targeting sequence (**Fig 5C**), the GFP-tagged *NDU1* localized to the *C*. *albicans* mitochondria, signifying that it may have functions in cellular respiration.

Loss of *NDU1* additionally lead to a striking reduction in *C*. *albicans* cell wall thickness due to prominent reduction in chitin and cell surface mannan content rendering them hypersusceptible to cell wall perturbing agents. This reduction in cell wall morphology was appreciated only after 48 h to 3 days of growth, around the time glucose was exhausted from the growth medium. As has been previously demonstrated by global gene expression profiling and biochemical studies, *C*. *albicans* cell wall and mannoprotein synthesis requires energy, most of which is provided through oxidative phosphorylation by the mitochondrial ETC [24–27]. Perhaps then, it was the defective growth phenotype of this mutant on alternative sources leading to cell wall alterations, which contributed toward the suboptimal biofilm phenotype. Inner layers of a mature biofilm are nutrient starved and hypoxic [28], and respiration is important for survival of these cells [29]. Premature detachment of biofilms formed by the mutant cells could prospectively be the consequence of loss of viability at the bottommost nutritionally disadvantaged layers of the biofilm, coupled with an early loss of adhesion to substrate due to alterations in the cell wall architecture. Analysis of expression levels of genes and proteins involved in viability or adhesion in the innermost cells of the mutant biofilms versus the WT biofilms will help understand the role of *NDU1* in maintaining the biofilm. Likewise, we have recently reported that when compared to planktonic or biofilm cells, biofilm-dispersed lateral yeast cells are metabolically rewired, expressing at significantly high levels, genes important for respiration, and nutrient assimilation [4]. Thus, loss of *NDU1* leading to reduced biofilm dispersal could be because of deficient respiratory activity, due to which energy required for production of new daughter cells from quiescent biofilm hyphae is lacking.

Energy in the cell is generated in the form of ATP, and Crabtree negative organisms such as *C*. *albicans* rely upon oxidation of substrates via the mitochondrial TCA cycle to generate ATP even in the presence of glucose [30,31]. In the classical respiratory pathway, complexes of the ETC (CI, CIII, and CIV) pump H^+^ ions to the intermembrane space. This creates a membrane potential (Δψ_Μ_) which is used by the ATP synthase to synthesize ATP [32]. Several lines of evidence including using Seahorse assays with CI substrates, in vitro activity and in situ gel analysis, measurement of mitochondrial membrane potential, and measurements of ROS were used to show that the major dysfunction of *NDU1* mutant was due to a defect in CI. A reduction in CI activity is harmful to the cells because it is the hub of all energy provided by oxidative phosphorylation and is the major ROS-generating unit in the mitochondria [13]. Dysfunction of CI destabilizes membrane potential and triggers ROS release, which is often fatal to the cell. *NDU1* null mutant was defective in both maintaining an intact membrane potential and keeping ROS under check.

*NDU1*’s shortcoming in utilizing alternative carbon sources and its disrupted cellular architecture caused mutant cells to be significantly more susceptible to primary human neutrophil cells. Indeed, innate immune cells are nutrient starved, and only those cells capable of growing under respiratory stress can combat these host cells [14]. In fact, *NDU1* mutant was strikingly avirulent in mice, with kidney organ burden of at least a log lower than mice infected with the WT strains. This defect in virulence in vivo was not due to a general growth defect of the mutant because between day 2 and day 5 of infection, the number of infected cells in the target organ, kidneys, increased by 5-fold in both mutant as well as the WT strain (**Fig 4D**). This virulence defect in the mutant was confirmed when *NDU1* expression levels were controlled in vivo (**Fig 4E**). It is well known that the integrity and function of mitochondria are essential to the virulence of *C*. *albicans*. Mutations affecting any one of a number of mitochondrial functions, including mitochondrial ribosome synthesis, mitochondrial transcription or genome maintenance, protein import, or functioning of the ETC, result in *C*. *albicans* virulence defect [12,26,27,33,34].

Our results on NDU1 and its role in the CI-related activity is reminiscent of another well-characterized CI protein in *C*. *albicans*, GOA1. *GOA1* deletion mutants fail to make CI, resulting in reduced respiration and multiple deficits on alternative carbon sources, cell wall alterations, enhanced sensitivity to killing by neutrophils, and reduced virulence in a murine model of disseminated disease [25,32,35,36]. However, the greatest difference between *NDU1* and *GOA1* is in their differential ability to undergo morphogenesis and form a biofilm. *GOA1* mutants cannot make hyphae or develop a biofilm [35], while *NDU1* is adept at both fronts. This indicates that *NDU1* might have regulatory functions different from *GOA1*. Interestingly, orthologues of *GOA1* are restricted to members of the “CTG clade” of fungi, whose members decode CTG codons as serine rather than leucine [35,37], suggesting that it represents a lineage-specific mitochondrial adaptation. Given that significant differences exist in CI among various lineages, and its important role in the pathobiology of *C*. *albicans*, an in-depth understanding of CI is warranted.

The *C*. *albicans* CI is believed to consist of 39 proteins, and while several of these proteins are conserved in other eukaryotes, a few such as GOA1 are unique to the CTG clade of which *Candida* species is a part [12, 36]. NDU1, however, has orthologues in other eukaryotes including humans. All fungal orthologues of NDU1 can be grouped into 1 clade and are monophyletic, meaning they inherited the gene from the same ancestor. The predicted structure of NDU1 is completely helical with a large central cavity that can accommodate 2 farnesyl pyrophosphate molecules. Three-dimensional modeling combined with bioinformatic and phylogenetic analysis revealed that NDU1 belongs to the family of dehydrosqualene synthases. Within this superfamily, NDU1 belongs to the family of Trans-Isoprenyl Diphosphate (Pyrophosphate) Synthases (Trans_IPPS; CDD: cd00867) and specifically the head-to-head family (Trans_IPPS_HH; CDD: cd00683) of synthases that catalyze the condensation of farnesyl or geranylgeranyl diphosphates to form squalene of cholesterol biosynthesis, or phytoene of carotenoid biosynthesis [38]. When a phylogenetic tree (adapted from [7]) with 50 Trans_IPPS domains was generated (**S6A Fig**), 3 monophyletic groups emerged: Cluster I proteins are putative NDU1 orthologues in fungi (green) and other eukaryotes and prokaryotes (pink); cluster 2 proteins that are phytoene synthases (PHYS) and squalene synthases (SQS); and cluster 3 which are the prenyl diphosphate synthases called COQ1 (coenzyme Q). The eukaryotic cluster 1 proteins from humans and drosophila have been experimentally localized to the mitochondrial inner membrane [39]. Likewise, NDU1 is predicted to be a peripheral membrane protein, embedded into the membrane via 5 amino acid residues of the carboxyl terminus.

NDU1 is 22% identical to its human orthologue NDUFAF6, with approximately 38% overall similarity between the 2 proteins. The Trans_IPPS_HH proteins typically have 2 DxxxD motifs involved in substrate binding and the coordination of catalytically important Mg++ ions [40]. NDU1, NDUFAF6, and their orthologues do not have these motifs, suggesting the possibility that they have lost the ability to function as squalene/phytoene synthases [7]. Besides, humans and other eukaryotes like *Candida* have a different and highly conserved functional squalene/phytoene synthase (ERG9) [41]. Thus, the actual function of NDU1-like proteins in eukaryotic mitochondria is unknown. The human orthologue NDUFAF6 has been demonstrated to play an important role in the assembly of CI through regulation of subunit ND1 biogenesis [39]. Future work using docking and molecular dynamics approaches combined with biochemical assays will be needed to understand if the NDU1 cavity can accommodate and bind isoprenoid ligands. The identity of NDU1 substrates and its catalytic activity associated with CI assembly is yet to be determined. However, the fact that NDU1 is predicted to be membrane localized could indicate that it interacts with amphiphilic or hydrophobic ligands or possess a chaperone-like role in assembling integral membrane subunit proteins of CI.

Despite overall similarities to human NDUFAF6, *C*. *albicans* NDU1 protein was found to be unique in its amino acid sequence. Compared to NDUFAF6, NDU1 is a significantly longer protein: 380 versus 333 amino acids (**Fig 5B**). The first long gap in NDU1 is part of the mitochondrial presequence; the human presequence is quite a bit longer than the *Candida* one. But the N-termini are removed upon import, so are not relevant to function. Note, however how several gaps have been introduced into the human sequence for the alignment to work because NDU1 is longer. The most compelling part of our study was that NDU1 protein sequence harbors 3 extra sets of amino acid inserts (**Fig 5C**), which are found only in CTG clade and very closely related fungi and missing from all other eukaryotes. One insert (insert 1) is positioned at the mouth of the cavity, while the other 2 inserts (insert 2 and 3) are structurally contiguous at the very bottom tip of the cavity (**Fig 6B**). Our gene deletion/complementation studies showed that insert 2, and 3, but not 1, are required for the function of NDU1, and for protecting cells from oxidative stress, by keeping ROS under check (**S6B Fig**). Because of the lack of insert 2 and 3 in the human NDUFAF6 orthologue, an overall low homology between the 2 proteins, and since NDU1 is required for virulence of *C*. *albicans*, the *C*. *albicans* NDU1 represents a highly desirable target for future novel therapeutic development to treat hematogenously disseminated candidiasis.

Inferring evolutionary mechanisms from genomic sequences with millions of years of divergence between them is inherently difficult. The idea that domain gains in eukaryotic proteins are directly mediated by gene duplication, followed by gene fusion and recombination may be the most plausible explanation. However, the *Candida* clade of species that are closely related to other Saccharomycotina did not undergo a whole genome duplication event [42]. Interestingly, small-scale duplication events did occur in *C*. *albicans*, and genomic diversity continues to increase during exposure to stress [43–45], thereby facilitating functional diversification and providing greater phenotypic flexibility [46–48]. Evolutionary diversity has also resulted in divergence in the posttranscriptional control of several processes in *Candida* spp. Specifically, mitochondrial protein synthesis and import is diverged in these fungi [12,49,50]. *C*. *albicans*, which last shared a common ancestor with *S*. *cerevisiae* at least 300 million years ago contains a complete ETC, while *S*. *cerevisiae* is devoid of CI (NADH: ubiquinone oxidoreductase) [51–53].

To conclude, our study reveals for the first time that following duplication, certain *C*. *albicans* genes may have acquired additional gene inserts to bolster protein–protein interactions. This unique evolutionary adaptation could also indicate lineage-specific changes in mitochondrial function, that likely are specific to how *Candida* adapts to nutritional stress. Why this selective acquisition of inserts is apparent only in NDU1 orthologues in the CTG clade fungi and not in any other eukaryotes is a subject worth investigating. Certainly, sequences in the NDU1 protein that are different from its human orthologue can be harnessed as targets, for small molecule compounds that can dock to it and abrogate function. Severe virulence defects in vivo upon mitochondrial dysfunction due to NDU1 deletion suggest that inhibition of this target would be an effective way to combat fungal infections. Indeed, presence of orthologues of NDU1 in the multidrug-resistant fungus *C*. *auris*, and in all other strains of *C*. *albicans* especially those resistant to antifungal drugs, means that mitochondrial inhibitors have a chance to act as pan-antifungal drugs.

## Methods

### Strains and culture conditions

Stock cultures of all strains were stored in 15% glycerol at −80°C. Strains were routinely grown under yeast conditions (media at 30°C) in YPG (1% yeast extract, 2% Bacto peptone, 2% glucose) or under filament-inducing conditions using RPMI medium (Sigma, St. Louis, Missouri, USA) with morpholinepropanesulfonic acid (MOPS) buffer. For experiments requiring alternative sources for growth, YP was supplemented with either 2% of potassium acetate, sorbitol, glycerol, or ethanol.

### Gene deletion and rescue of orf9.2500 (*NDU1*)

To generate orf19.2500 mutant, the orf19.2500 was replaced with the deletion cassette containing URA3 gene [54] as a selectable marker gene flanked with fragments corresponding to 500-bp upstream and downstream flanking sequences of the orf19.2500. We added KpnI and XhoI restriction sites to the ends of upstream fragment and NotI and SacII restrictions sites to the downstream fragment by PCR for cloning. The deletion cassette was released by KpnI and SacII restriction enzymes and transformed into BWP17 cells [55] followed by spreading the cells on uracil dropout medium. The heterozygous strain was confirmed by PCR and subjected to another round of gene deletion using a deletion cassette containing ARG4 as a selectable marker to prepare null mutant strain.

To complement orf19.2500 mutant, we generated a complemented cassette containing a full length of orf19.2500, the nourseothricin resistance gene as a selectable marker and 500 bp of the terminator region of the orf19.2500 gene using pJK890 [55]. The ORF19.2500 sequence was cloned with KpnI and ApaI restriction enzymes and the downstream part was cloned with NotI and SacII restriction enzymes into pJK890. The rescue cassette was released by KpnI and SacII restriction enzymes and transformed into the orf19.2500 mutant strain. The cells were spread on YPD containing 200 ug/ml nourseothricin as a selection medium. The correct transformants were screened by PCR. To rescue the second allele of the ORF19.200 gene in this heterozygous strain, the nourseothricin resistance gene was looped out from the cells as described previously [6]. Further, the cells were subjected to the rescue cassette again to receive the second allele of ORF19.2500 gene. The homozygous strain was confirmed by PCR.

### GFP and mCherry tagging combined with regulation of expression

We overexpressed orf19.2500 under Tetoff promoter and tagged the gene with GFP or mCherry at carboxyl terminus. To do so, first the selective marker URA3 was replaced with ARG4 in pGS1245 (Tetoff-GFP-TetR-URA3) [56], then the full-length sequence of orf19.2500 was integrated into the plasmid via XhoI restriction enzyme. The plasmid was digested with AscI and transformed into orf19.2500−/− mutant to produce overexpressed strain. To tag the gene with mCherry, the GFP was replaced with mCherry sequence with XhoI and ClaI restriction enzymes. A similar approach was used also to overexpress the Tet-O promoter driven GFP-tagged, full-length sequence of the human gene NDUFAF6 or the individual NDU1 insertion sequences in *C*. *albicans* orf19.2500 mutant strain.

### Growth rate determination

For cell dilutions spotted onto agar media as previously described [4], saturated overnight cultures were diluted in 4- to 5-fold steps from an OD_600_ of 0.5. The stressors used were YPG agar plus 50 μg/ml calcofluor white, or 10 μg/ml congo red or 0.025% SDS. For growth curves in liquid media, saturated overnight cultures in YPD were washed once in 0.9% NaCl and diluted to an OD_600_ of 0.15 in 150 μL medium in flat-bottomed 96-well dishes. For growth assays, OD_600_ readings were obtained every 60 min in a plate reader, and SDs of 3 technical replicates were calculated and graphed. For viability counts, *C*. *albicans* strains were inoculated at a concentration of 1 × 10^6^ cells/ml in 250 ml YPG medium. Every day up to 15 days, an aliquot of cells were recovered, diluted, counted using a hemocytometer, and plated on YPG agar plates. Colonies were counted, calculated, and plotted, representing the viability of the cells over time.

### Biofilm growth and dispersal

Biofilms were grown both under static and flow conditions. For static growth, 1 ml of *C*. *albicans* cells (1 × 10^6^ cells/ml) was added to the wells of a 24-well microtiter plate and incubated overnight in RPMI, and the biofilms were gently washed 2 times [57]. These biofilms were quantified using the XTT assay [57], at various time points of growth. For enumeration of dispersed cells, static biofilm supernatants were collected after 24 h of growth, and turbidity (OD_600_) was measured by a spectrophotometer. For growth under the flow system, biofilms were developed on SE material, as previously described [58]. At various times during biofilm growth, media flowing over the biofilms were collected, and biofilm-dispersed cells present in the media were counted using a hemocytometer and plotted. Extent of attachment of the biofilm to its substrate was examined by gentle washing of the biofilm in the static model or teasing the biofilm away from the substrate using a sterile needle. A small aliquot of the biofilm hyphae were also visualized under a phase contrast microscope (40× mag) to appreciate the extent of hyphae to lateral yeast growth.

### Assessment of phenotypic properties

#### Damage to HUVEC

Human umbilical cord endothelial cells (HUVEC) were isolated following an established protocol [3]. The ability of *C*. *albicans* to damage human vascular endothelial cells was assessed by the CytoTox-96 assay (Promega, Madison, Wisconsin, USA), which measures the release of lactate dehydrogenase (LDH) from dying cells. For these experiments, WT and mutant cells were diluted to various concentrations in HUVEC culture medium and were added to endothelial cells for 16-h incubation times at 37°C in the presence of 5% CO2. The amount of LDH released from the coculture system was quantified by spectrophotometry. Uninfected cultures (control 1) and *C*. *albicans* alone (control 2) incubated under identical conditions were included as negative controls. The total amount of LDH released was estimated by treating control uninfected endothelial cells with 9% Triton X-100 for 1 hour. The LDH released in the presence of *C*. *albicans* was quantified by using the following formula: [(experimental − control 1 − control 2)/(total − control 1)] ×100. The values were expressed as percentages of the total amount of LDH released.

#### Cell membrane permeability

*Candida* strains were grown in YPG for 48 h and about 5 × 10^6^ cells were resuspended and washed twice in 1 ml of FDA buffer before supplementing with 50 nm FDA. A 200-μl volume of cell mixture with or without FDA was added to an optical-bottom 96-well plate. The kinetics of FDA uptake was recorded every 5 min for 30 reads with simultaneous shaking of samples in a plate reader with an excitation and emission wavelengths 485 and 535 nm, respectively. Data represent the fluorescence intensity over time.

#### Flow cytometry for cell component analysis

To stain mannan and chitin of the cell wall, *C*. *albicans* yeast cells grown for 48 h were washed in PBS and incubated in the dark with 25 μg/ml Concanavalin A to stain for α-mannopyranosyl or 5 μg/ml CFW for chitin for 30 min. The above stained cells were washed, fixed, and differences intensity of the staining measured by flow cytometer at approximately 495/519 nm or 380/475 nm, respectively.

#### Transmission electron microscopy

*C*. *albicans* cells grown for 48 h were washed in PBS and then fixed in 4-ml fixative solution (3% paraformaldehyde, 2.5% glutaraldehyde, pH 7.2) for 24 h at 4°C. After postfixation of samples with 1% phosphotungstic acid for 2 h, they were washed by distilled water, block-stained with uranyl acetate, dehydrated in alcohol, immersed in propylenoxide, and embedded in glycide-ether. Ultrathin sections were observed under a JEOL 100CX transmission electron microscope.

#### Fluconazole susceptibility

Fluconazole activity was assessed by Epsilometer test strips (Etest strips) (bioMérieux, St. Louis, Missouri, USA) according to the manufacturer’s instructions. A standardized cell suspension (a 0.5 McFarland standard) was used to create a confluent lawn across YPD agar plates prior to Etest strip placement, and the cells were then incubated at 30°C for 48 h.

#### Glucose replenishment assay

Overnight cultures of WT, NDU1 mutant, and revertant strains were subcultured in 5 ml of YP+ 1% Acetate (1 × 10^5^ cells/ml) and incubated at 30°C for 24 h. After incubation, the cultures were supplemented with glucose to a final concentration of 1% and re-incubated further for 24 h. Turbidity of the broth cultures were monitored visually.

WT and mutant cells (1 × 10^5^ cells) were also spread on a lawn of YP+ 1% Acetate solid medium and incubated for 4 h at 30°C. Sterile filter paper discs soaked in 2% glucose were then placed on the respective media surface and plates incubated for 24 h. Colony growth on the solid media were monitored visually.

### Neutrophil killing

After obtaining institutional review board–approved consent (The Lundquist protocol # 11672–07), neutrophils were isolated from blood collected from human volunteers using endotoxin-free Ficoll-Paque Plus reagent (Amersham Biosciences, Piscataway, New Jersey, USA). The killing assay was carried out as described previously [59]. Briefly, neutrophils were incubated with *C*. *albicans* yeast cells (neutrophil:fungus ratio, 3:1). Controls contained *C*. *albicans* without neutrophils. After 150 min, the mixtures were sonicated to disrupt neutrophils and the surviving fungi quantitatively cultured. The percentage of opsonophagocytic killing (OPK) was calculated by dividing the number of colony-forming unit (CFU) in the tubes containing neutrophils by the number of CFU in tubes without neutrophils. *C*. *albicans* phagocytosis by neutrophils were visualized at 90 and 150 min, using a phase contrast microscope (40× mag).

### Virulence assays

Animal studies were approved by the IACUC of The Lundquist Institute at Harbor–UCLA Medical Center, according to the NIH guidelines for animal housing and care. For the *C*. *albicans* infection in vivo, groups of CD1 female mice (6 to 8 weeks) were injected via lateral tail vein with 200 μl of a suspension containing indicated live *C*. *albicans* (2.5 × 10^5^ cells or 2.5 × 10^6^ cells) in sterile saline. Mice were monitored daily and differences in survival between infected groups were compared by the log-rank test. Quantitative culturing of kidneys from mice infected with different strains of *Candida* was performed; mice were infected through tail veins, kidneys were harvested 2 and 5 days post infection, homogenized, serially diluted in 0.85% saline, and quantitatively cultured on YPG that contained 50 μg/ml chloramphenicol. Colonies were counted after incubation of the plates at 37°C for 24 to 48 hr, and results were expressed as log CFU per gram of infected organ.

Virulence assay under regulated gene expression conditions in vivo: Cultures of *C*. *albicans* strains for injection were grown overnight in YPD medium without DOX and incubated at 30°C. Cells (2.5 × 10^5^ cells in 200 μl of pyrogen-free saline solution per mouse) of the *C*. *albicans* tetO-NDU1/ndu1 strain were delivered by tail vein injection into 2 groups of mice, each consisting of eight 6- to 8-week-old female CD1 mice, with or without DOX in their drinking water (2 mg/ml in 5% sucrose). Cells of the control NDU1/NDU1 strain were injected at the same infecting dose into another group of animals (*n* = 8) with DOX in their drinking water. Pathogenicity of WT strains not containing any tetracycline-regulatable element is not affected by the presence or absence of DOX [60]. Mice were monitored daily and differences in survival between infected groups were compared by the log-rank test.

### Mitochondria associated assays

#### Sphaeroplast and mitochondria preparations

Cells were grown in 250 ml of YPD broth overnight at 30°C, washed once with cold water and once with buffer A (1 M sorbitol, 10 mM MgCl2, 50 mM Tris-HCl [pH 7.8]), centrifuged (5,000 rpm for 10 min). Cells were suspended in buffer A (50 ml) plus 30 mM dithiothreitol (DTT) for 15 min at 30°C with shaking (100 rpm) and then collected and suspended in buffer A with 1 mM DTT plus 100 mg of Zymolyase 20T (MP Biomedicals) per 10 g of pelleted cells. Shake cultures (100 rpm) were incubated at 30°C for 60 min or until 90% of cells were converted into spheroplasts (as determined by light microscopy). Spheroplasts were washed twice with buffer A. Crude preparations of mitochondria were isolated as previously described [32]. Briefly, spheroplasts were suspended in 10 ml of cold buffer B (0.6 M mannitol, 1 mM EDTA, 0.5% bovine serum albumin [BSA], 1 mM phenylmethylsulfonyl fluoride [PMSF], 10 mM Tris-HCl [pH, 7.4]) and then broken mechanically using a Dounce homogenizer on an ice bath. Cell debris was removed by low-speed centrifugation (1,000 X g for 10 min). The supernatants containing mitochondria were centrifuged at 10,500 X g for 10 min, and the pellet was washed twice with 20 ml of ice-cold buffer C (0.6 M mannitol, 1 mM EDTA, 1% BSA, 10 mM Tris-HCl [pH 7.0]). Mitochondria were suspended in 1 ml of buffer D (0.6 M mannitol, 10 mM Tris-HCl, [pH 7.0]), and the protein content was determined by Bradford method.

#### Blue native PAGE

Mitochondrial protein was concentrated by vacuum centrifugation. Ten microliters of BN sample buffer (2X) was mixed with 20 μl of each sample (60 to 80 μg of protein) and loaded onto a BN-PAGE gradient gel (4 to 16%) (Invitrogen, Carlsbad, California, USA). One ml of 2X BN sample buffer consisted of 1.5 M 6-aminohexanoic acid, 0.05 M bis-Tris (pH 7.0), 65 μl of 10% DMM, 20 μl of proteinase inhibitor mixture, and 100 μl of glycerol. Electrophoresis was performed in an X-Cell SureLock mini-cell system (Invitrogen) with 200 ml of cathode buffer in the upper (inner) buffer chamber and 150 ml of anode buffer in the lower (outer) buffer chamber. Electrophoresis was carried out at 4°C and 65 V for 1 h and then raised to 120 V overnight. An in-gel enzyme assay for CRC CI was accomplished as follows: Gels were rinsed briefly twice with MilliQ water and equilibrated in 0.1 M Tris-HCl, pH 7.4 (reaction buffer), for 20 min. The gels were then incubated in fresh reaction buffer with 0.2 mM NADH–0.2% nitroblue tetrazolium (NBT) for 1 h. Reactions were stopped by fixing the gels in 45% methanol–10% (vol/vol) acetic acid, and then gels were destained overnight in the same solution. Image processing of gels was done using ImageJ software.

#### Enzymatic assay of CI

Mitochondrial protein was dissolved in 0.8 ml sterile water and incubated for 2 min at 37°C, then mixed with 0.2 ml of a solution containing 50 mM Tris pH 8.0, 5 mg /ml BSA, 0.24 mM KCN, 4 μM antimycin A, and 0.8 mM NADH, the substrate for CI. The reaction was initiated by introducing an electron acceptor, 50 μM DB (2,3-dimethoxy-5-methyl-6-n-decyl-1,4 benzoquinone). Enzyme activity was followed by a decrease in absorbance of NADH at 340 nm minus that at 380 nm using an extinction coefficient of 5.5 mM^−1^cm^−1^.

#### ROS measurement

Intracellular ROS production was detected by staining cells with 5 μM MitoSOX Red (Life Technologies, Frederick, Maryland, USA) in DMSO. Cells from 25-ml cultures grown at 30°C overnight in YPD medium were collected and washed twice with PBS. The pellets were suspended to 1 × 10^6^ cells in 1 ml of PBS and treated with or without MitoSOX Red for 45 min at 30°C in the dark. Cell fluorescence in the presence of DMSO alone was used to verify that background fluorescence was similar per strain. Cells from each MitoSOX-treated sample were collected and washed twice with PBS after staining, and mean fluorescence for ROS was quantified.

#### Oxygen consumption rate assay

OCR were measured under a Seahorse instrument (Seahorse Bioscience, Massachusetts, USA) according to the manufacturer’s instructions. Isolated mitochondria from overnight grown WT, mutant, and revertant cells were seeded into wells of a poly-d-lysine-coated XF96 spheroid plate containing 100 μL/well of warm assay medium (Seahorse XF base medium minimal DMEM, supplemented with 3 mM glucose and 0.1% FBS). A total of 25 μl of mitochondrial suspension, containing 3 μg of protein for the succinate condition and pyruvate/malate condition, were added to a Seahorse 96-well plate and centrifuged (2000 g × 20 min × 4°C). After centrifugation, 155-μl assay buffer containing pyruvate (10 mM) in combination with malate (2 mM) or succinate (10 mM) and rotenone (2 μM) (all final concentrations and pH 7.2) were added, and the plate was analyzed at 37°C. Absolute OCR is presented as pmol O2 consumed/min/μg protein. Mitochondrial OCR was determined by subtracting the antimycin A (1 μM, Sigma) and Rotenone (1 μM, Sigma)-sensitive OCR from the post-treatment OCR. Basal respiration was calculated in the presence of respiratory substrates (before ADP addition). Percentage inhibition was determined by dividing the post-treatment OCR with the basal mitochondrial OCR (antimycin A and Rotenone corrected) [61].

#### Mitochondrial membrane potential assay

The mitochondrial inner membrane potential (Δψm) was determined by staining with the membrane-permeable lipophilic cationic fluorochrome JC-1 (BD Biosciences, New Jersey, USA). Overnight, *C*. *albicans* cultures were washed, diluted to 1 × 10^6^ cells/ml of PBS, treated with JC-1 (3 μM final concentration), and incubated at 37°C for 30 min. Cells were washed and resuspended in 1 ml PBS and fluorescence dye accumulation measured using a flow cytometer equipped with a 488-nm argon excitation laser and 525-nm emission, and bandpass filters designed to detect green FITC dye [62].

## Supporting information

S1 Fig (A) Biofilms of WT, mutant (−/−), and revertant (+/+) strains were developed under the flow biofilm system [58] for 24 h using YNB medium. Dispersal from biofilms were measured at various time points (3 biological replicates) using a hemocytometer, as described earlier [3]. (B) Topmost layers of the biofilm were teased at 16 h to reveal extent of lateral yeast growth from WT, −/−, or +/+ biofilms. Scale bar represents 10 μm. (C) Growth curve of WT vs. orf19.2500 mutant over 16 days (3 replicates each time point). Cells were grown in YP+2% glucose broth for approximately 2 weeks. At various time points mentioned in the figure, aliquots of culture were measured for viability on solid YPD media. Blue curve = WT while red curve = mutant (D). Colony size of mutant versus WT after 4 days of growth (E). Hyphal lengths of WT and mutant compared visually (F). Damage caused by WT and mutant cells to HUVEC cells measured after 24 h using the LDH assay as described previously[3] (G). Left panel: WT, mutant, and revertant strains were grown in YP+1% acetate for 24 h after which glucose was added to the tubes at 1% final concentration. While mutant strain did not grow in 1% acetate, a resurgence of growth was seen once glucose was added. WT and revertant strains grew equally well regardless of the carbon source. Right panel: WT or mutant (−/−) cells were plated on solid YP media containing 1% acetate for 4 h, after which sterile filter paper discs containing 2% glucose were placed on the plates, and incubated for 24 h. While WT grew everywhere on the plate, mutant (−/−) strains could only grow around the glucose discs, Raw data are found in the file S1 Data. HUVEC, human umbilical cord endothelial cell; LDH, lactate dehydrogenase; WT, wild-type.
(TIFF)

S2 Fig (A) Estimation of cell surface mannan and chitin content by staining WT, mutant, and revertant with ConA and calcofluor white, respectively, and measured by flow cytometry. (B) Visualization and measurement of the differences in thickness of the cell wall structure between WT and mutant cells, by transmission electron microscopy. The histogram shows difference in cell wall thickness between WT and −/− cells (* = *p* < 0.01) (C). Quantitation of the extent of cell membrane permeability between WT and orf19.2500 mutant, in the presence and absence of fluconazole (D). Quantitation of the expression of ergosterol genes in WT and mutant cells. Raw data are found in the file S1 Data. WT, wild-type.
(TIFF)

S3 Fig (A) Measurement of OCRs of mitochondria isolated from WT, mutant, and revertant strains, in presence of CII substrates succinate+rotenone (B). Determination on defect in mitochondrial membrane integrity in the WT, mutant and revertant strain, on growth in glucose or acetate, by using the JC1 dye (C). Survival of mice infected with a 10-fold higher infection dose of 2.5 × 10^6^ cells, of WT mutant and revertant cells. Raw data are found in the file S1 Data. CII, Complex II; OCR, oxygen consumption rate; WT, wild-type.
(TIFF)

S4 Fig (A) Surface display of 2 FPS bound in the large pocket in 5iys from *E*. *hirae*. Mg^++^ (green spheres), water molecules (red spheres) (B). Surface display of the pocket of c5iysA model while still showing FPS as they are positioned in 5iys. Note, while the pocket is in a different shape and the substrates cannot bind in the same orientations, the pocket is large enough to accommodate the 2 FPS. (C) Model predicted by Phyre2 shows c4hd1A (green), which is NDU1 modeled on 4hd1, a squalene synthase from *A*. *acidocaldarius* (D). Red highlighting of the identical residues between NDU1 and human NDUFAF6 (gray). (E) A phylogenetic tree was formulated based on the presence or absence of the 3 insertion sequences (IS1, IS2, and IS3) in eukaryotes. The tree clearly divided into 3 groups: group 1 colored blue contained Saccharomycetales and *Candida* like CTG clade fungi (CTG clade), group 2 in black which had other fungi, and group 3 colored pink represented sequences from bacteria and eukaryotes. Group 1 had longer branches and were well separated from group 2 and 3 sequences. Also, only group 1 and exclusively the CTG clade yeasts had all 3 insert sequences. In contrast, group 2 had no insert 1 or 2 and had a different insert 3, while group 3 were lacking in all the inserts. FPS, farnesyl thiopyrophosphate.
(TIFF)

S5 Fig (A) Expression and localization of *C*. *albicans* NDU1 without inserts. Entire ORF of GFP-tagged NDU1 without individual inserts were expressed separately in *C*. *albicans* NDU1 mutant, and found to be localized to mitochondria, as visualized by GFP overlapping with a mitochondrial stain. I1, I2, and I3 stand for NDU1 expressed with truncations in insert 1, 2, and 3, respectively. Scale bar for top and bottom panels 2 μM. (B) Detection of the GFP-tagged NDU1 protein without individual inserts by western blotting using an anti-GFP antibody. M = marker, second lane is 65.1 kDa NDU1 minus insert 1, lane 2 is 64.7 kDa NDU1 minus insert 2, lane 3 is 63.5 kDa NDU1 minus insert 1. Raw data are found in the file S1 Raw Images. (C) Structural model of the interaction of NDU1 with the surface of the inner mitochondrial membrane.
(TIFF)

S6 Fig (A) Phylogeny of NDU1. NDU1 belongs to the Trans_IPPS family. Proteins were aligned using TCOFFEE. Support values for nodes are from MrBayes (upper value) and RAxML (lower value). Putative orthologs of NDU1 form Cluster 1; PHYS and SQS homologs form Cluster 2 and COQ1 (coenzyme Q1 synthase, decaprenyl diphosphate synthase) homologs form Cluster 3. S6B Flow cytometry data of *C*. *albicans* strains stained with MitoSox Red, an indicator of ROS activity. ROS production in NDU1 mutant overexpressing NDU1 without respective inserts were compared to ROS activity in WT and mutant strains. *p* < 0.01 of the indicated conditions versus WT. Raw data are found in the file S1 Data. PHYS, phytoene synthase; ROS, reactive oxygen species; SQS, squalene synthase; WT, wild-type.
(TIFF)

S1 Data This file contains numerical values for figures as indicated in the individual sheets. (XLSX)

S1 Raw Images This file includes the original gel images for the indicated figures. (TIFF)

## Abbreviations

3D: three-dimensional
BN-PAGE: Blue native PAGE
BSA: bovine serum albumin
CFU: colony-forming unit
CI: Complex I
CII: Complex II
DOX: doxycycline
ETC: electron transport chain
FDA: fluorescein diacetate
FGSC: Fungal Genetics Stock Center
FPS: farnesyl thiopyrophosphate
HUVEC: human umbilical cord endothelial cell
LDH: lactate dehydrogenase
mNDU1: mature NDU1
MOPS: morpholinepropanesulfonic acid
OCR: oxygen consumption rate
OPK: opsonophagocytic killing
OPM: Orientations of Proteins in Membranes
PHYS: phytoene synthase
PMSF: phenylmethylsulfonyl fluoride
ROS: reactive oxygen species
SE: silicone elastomer
SQS: squalene synthase
TCA: tricarboxylic acid
WT: wild-type
