# Supplementary figures and images for: An evolutionarily diverged mitochondrial protein controls biofilm growth and virulence in Candida albicans
Source: PLoS Biol. 2021 Mar 15;19(3):e3000957. doi: 10.1371/journal.pbio.3000957 (PMC8007014; doi:10.1371/journal.pbio.3000957)

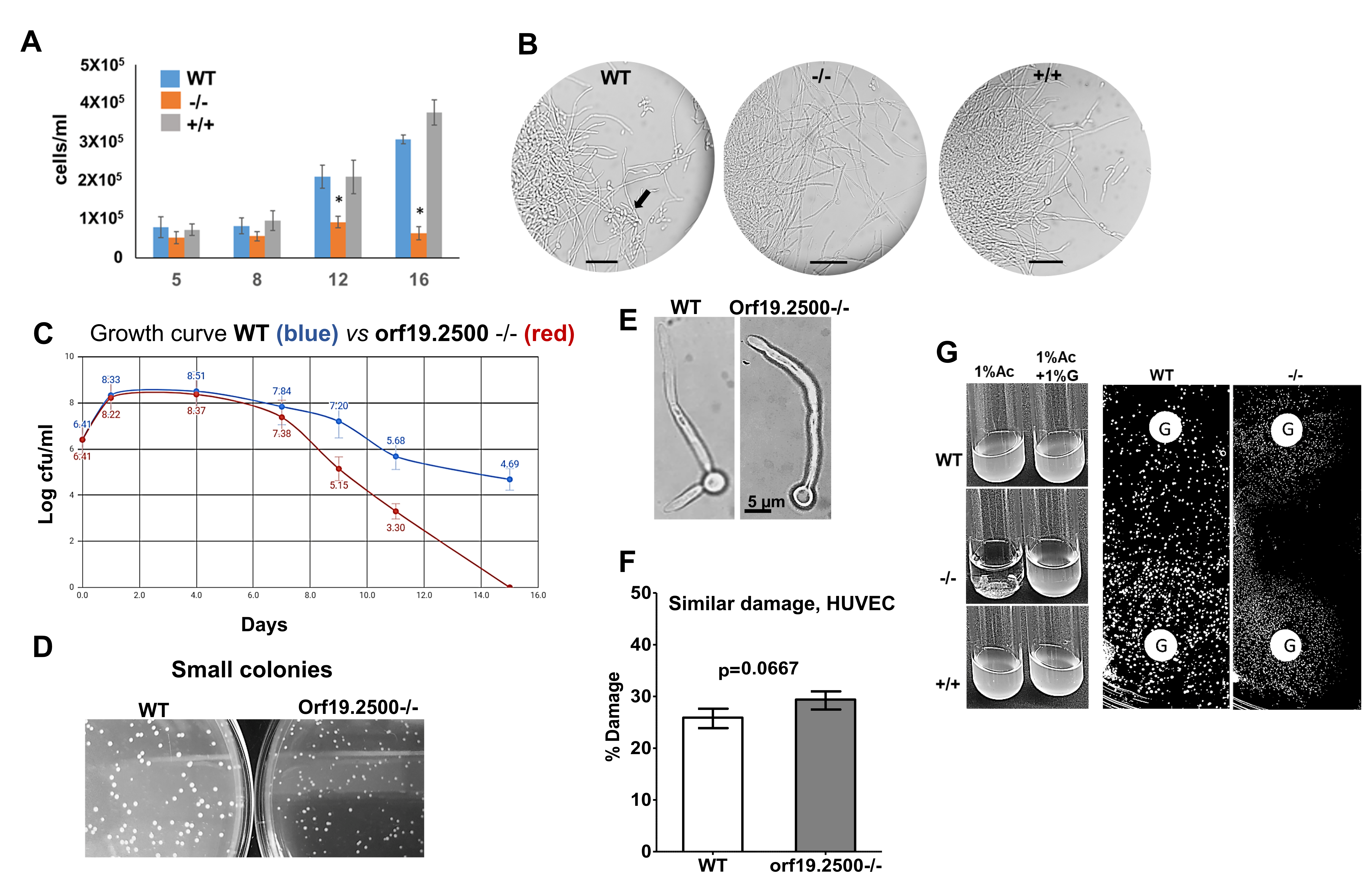

Supplement: S1 Fig — (A) Biofilms of WT, mutant (−/−), and revertant (+/+) strains were developed under the flow biofilm system [58] for 24 h using YNB medium. Dispersal from biofilms were measured at various time points (3 biological replicates) using a hemocytometer, as described earlier [3]. (B) Topmost layers of the biofilm were teased at 16 h to reveal extent of lateral yeast growth from WT, −/−, or +/+ biofilms. Scale bar represents 10 μm. (C) Growth curve of WT vs. orf19.2500 mutant over 16 days (3 replicates each time point). Cells were grown in YP+2% glucose broth for approximately 2 weeks. At various time points mentioned in the figure, aliquots of culture were measured for viability on solid YPD media. Blue curve = WT while red curve = mutant (D). Colony size of mutant versus WT after 4 days of growth (E). Hyphal lengths of WT and mutant compared visually (F). Damage caused by WT and mutant cells to HUVEC cells measured after 24 h using the LDH assay as described previously[3] (G). Left panel: WT, mutant, and revertant strains were grown in YP+1% acetate for 24 h after which glucose was added to the tubes at 1% final concentration. While mutant strain did not grow in 1% acetate, a resurgence of growth was seen once glucose was added. WT and revertant strains grew equally well regardless of the carbon source. Right panel: WT or mutant (−/−) cells were plated on solid YP media containing 1% acetate for 4 h, after which sterile filter paper discs containing 2% glucose were placed on the plates, and incubated for 24 h. While WT grew everywhere on the plate, mutant (−/−) strains could only grow around the glucose discs, Raw data are found in the file S1 Data. HUVEC, human umbilical cord endothelial cell; LDH, lactate dehydrogenase; WT, wild-type. (TIFF) [file pbio.3000957.s001.tiff]

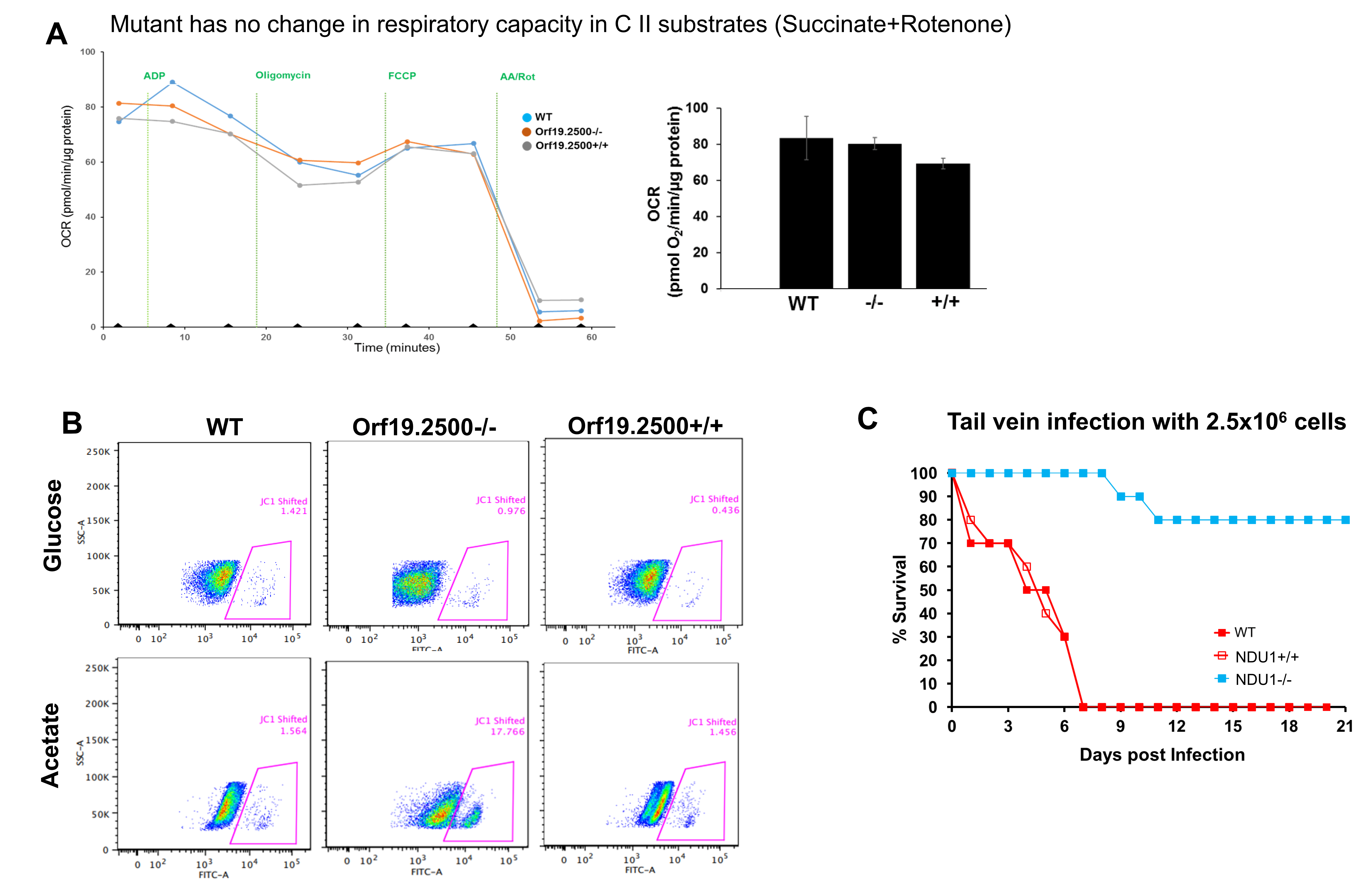

Supplement: S3 Fig — (A) Measurement of OCRs of mitochondria isolated from WT, mutant, and revertant strains, in presence of CII substrates succinate+rotenone (B). Determination on defect in mitochondrial membrane integrity in the WT, mutant and revertant strain, on growth in glucose or acetate, by using the JC1 dye (C). Survival of mice infected with a 10-fold higher infection dose of 2.5 × 10^6^ cells, of WT mutant and revertant cells. Raw data are found in the file S1 Data. CII, Complex II; OCR, oxygen consumption rate; WT, wild-type. (TIFF) [file pbio.3000957.s003.tiff]

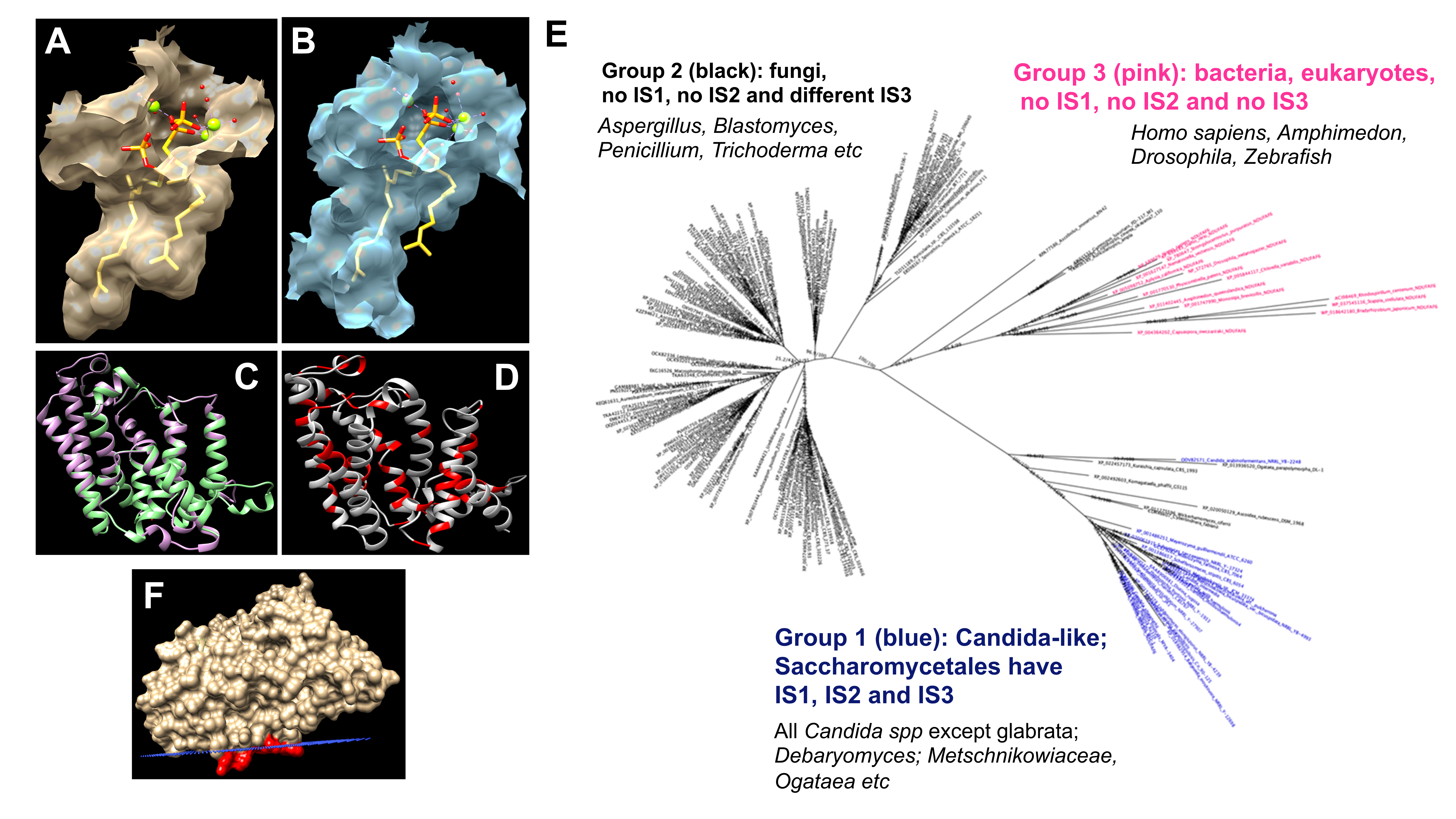

Supplement: S4 Fig — (A) Surface display of 2 FPS bound in the large pocket in 5iys from E. hirae. Mg++ (green spheres), water molecules (red spheres) (B). Surface display of the pocket of c5iysA model while still showing FPS as they are positioned in 5iys. Note, while the pocket is in a different shape and the substrates cannot bind in the same orientations, the pocket is large enough to accommodate the 2 FPS. (C) Model predicted by Phyre2 shows c4hd1A (green), which is NDU1 modeled on 4hd1, a squalene synthase from A. acidocaldarius (D). Red highlighting of the identical residues between NDU1 and human NDUFAF6 (gray). (E) A phylogenetic tree was formulated based on the presence or absence of the 3 insertion sequences (IS1, IS2, and IS3) in eukaryotes. The tree clearly divided into 3 groups: group 1 colored blue contained Saccharomycetales and Candida like CTG clade fungi (CTG clade), group 2 in black which had other fungi, and group 3 colored pink represented sequences from bacteria and eukaryotes. Group 1 had longer branches and were well separated from group 2 and 3 sequences. Also, only group 1 and exclusively the CTG clade yeasts had all 3 insert sequences. In contrast, group 2 had no insert 1 or 2 and had a different insert 3, while group 3 were lacking in all the inserts. FPS, farnesyl thiopyrophosphate. (TIFF) [file pbio.3000957.s004.tiff]

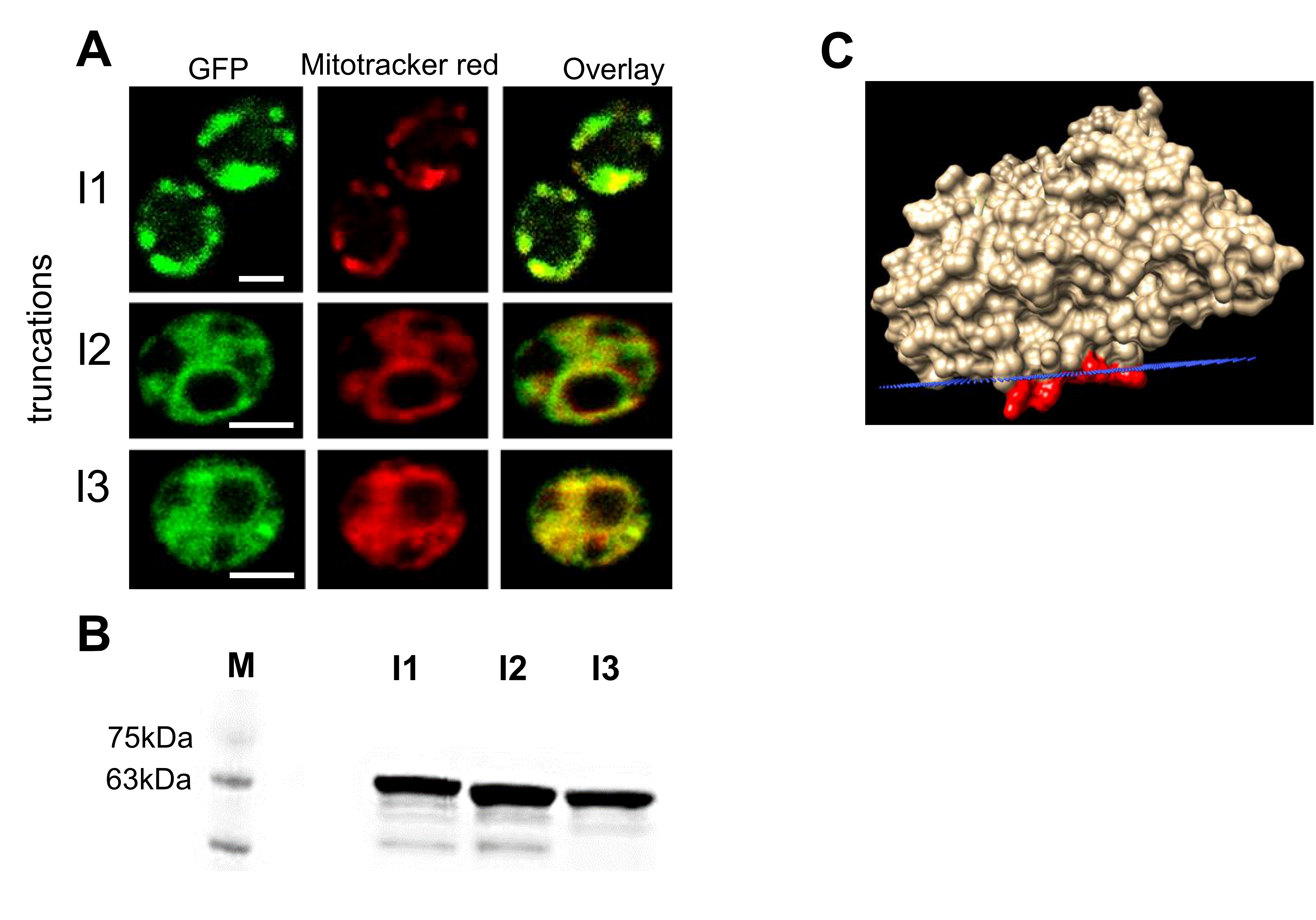

Supplement: S5 Fig — (A) Expression and localization of C. albicans NDU1 without inserts. Entire ORF of GFP-tagged NDU1 without individual inserts were expressed separately in C. albicans NDU1 mutant, and found to be localized to mitochondria, as visualized by GFP overlapping with a mitochondrial stain. I1, I2, and I3 stand for NDU1 expressed with truncations in insert 1, 2, and 3, respectively. Scale bar for top and bottom panels 2 μM. (B) Detection of the GFP-tagged NDU1 protein without individual inserts by western blotting using an anti-GFP antibody. M = marker, second lane is 65.1 kDa NDU1 minus insert 1, lane 2 is 64.7 kDa NDU1 minus insert 2, lane 3 is 63.5 kDa NDU1 minus insert 1. Raw data are found in the file S1 Raw Images. (C) Structural model of the interaction of NDU1 with the surface of the inner mitochondrial membrane. (TIFF) [file pbio.3000957.s005.tiff]

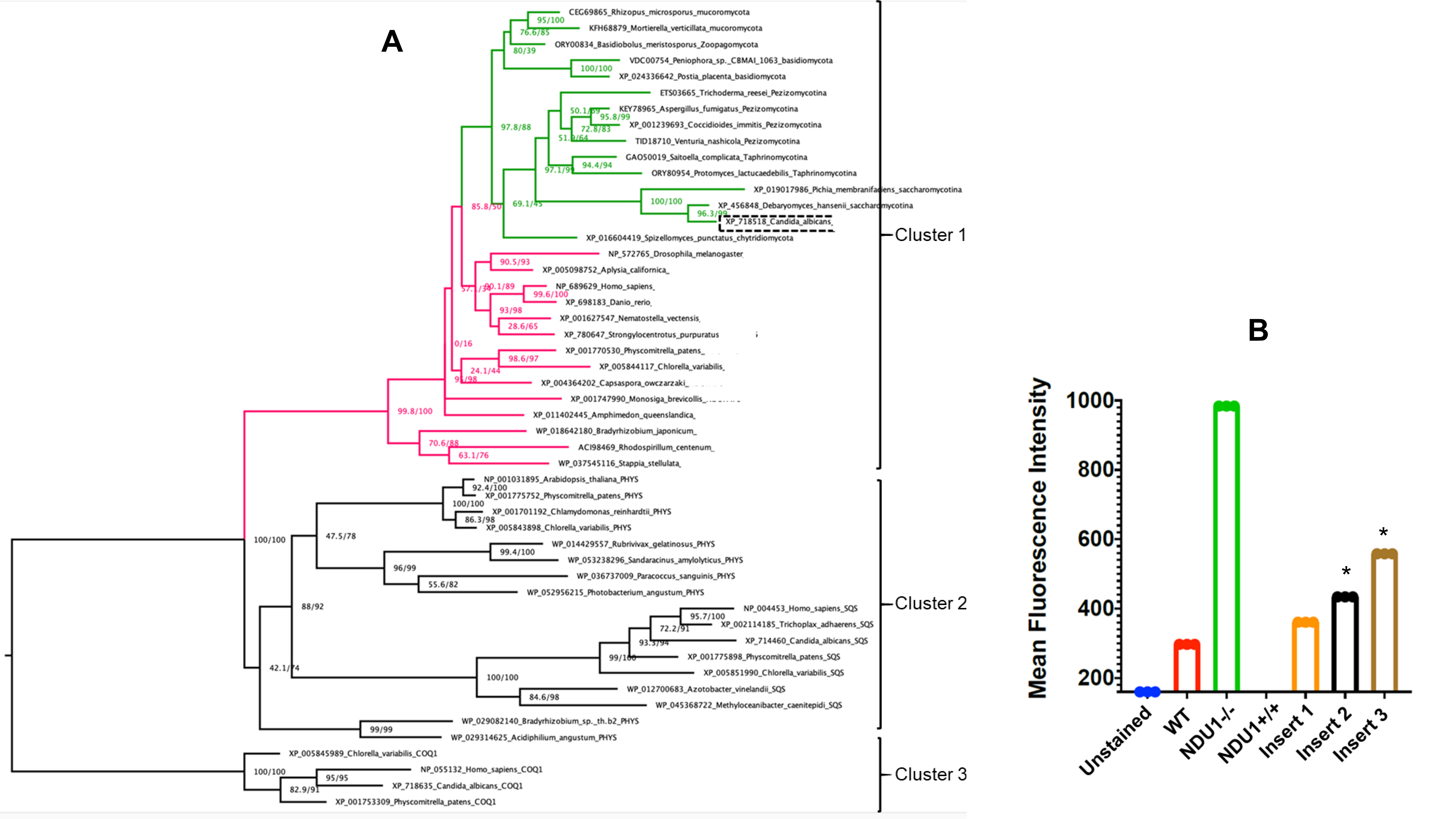

Supplement: S6 Fig — (A) Phylogeny of NDU1. NDU1 belongs to the Trans_IPPS family. Proteins were aligned using TCOFFEE. Support values for nodes are from MrBayes (upper value) and RAxML (lower value). Putative orthologs of NDU1 form Cluster 1; PHYS and SQS homologs form Cluster 2 and COQ1 (coenzyme Q1 synthase, decaprenyl diphosphate synthase) homologs form Cluster 3. S6B Flow cytometry data of C. albicans strains stained with MitoSox Red, an indicator of ROS activity. ROS production in NDU1 mutant overexpressing NDU1 without respective inserts were compared to ROS activity in WT and mutant strains. p < 0.01 of the indicated conditions versus WT. Raw data are found in the file S1 Data. PHYS, phytoene synthase; ROS, reactive oxygen species; SQS, squalene synthase; WT, wild-type. (TIFF) [file pbio.3000957.s006.tiff]

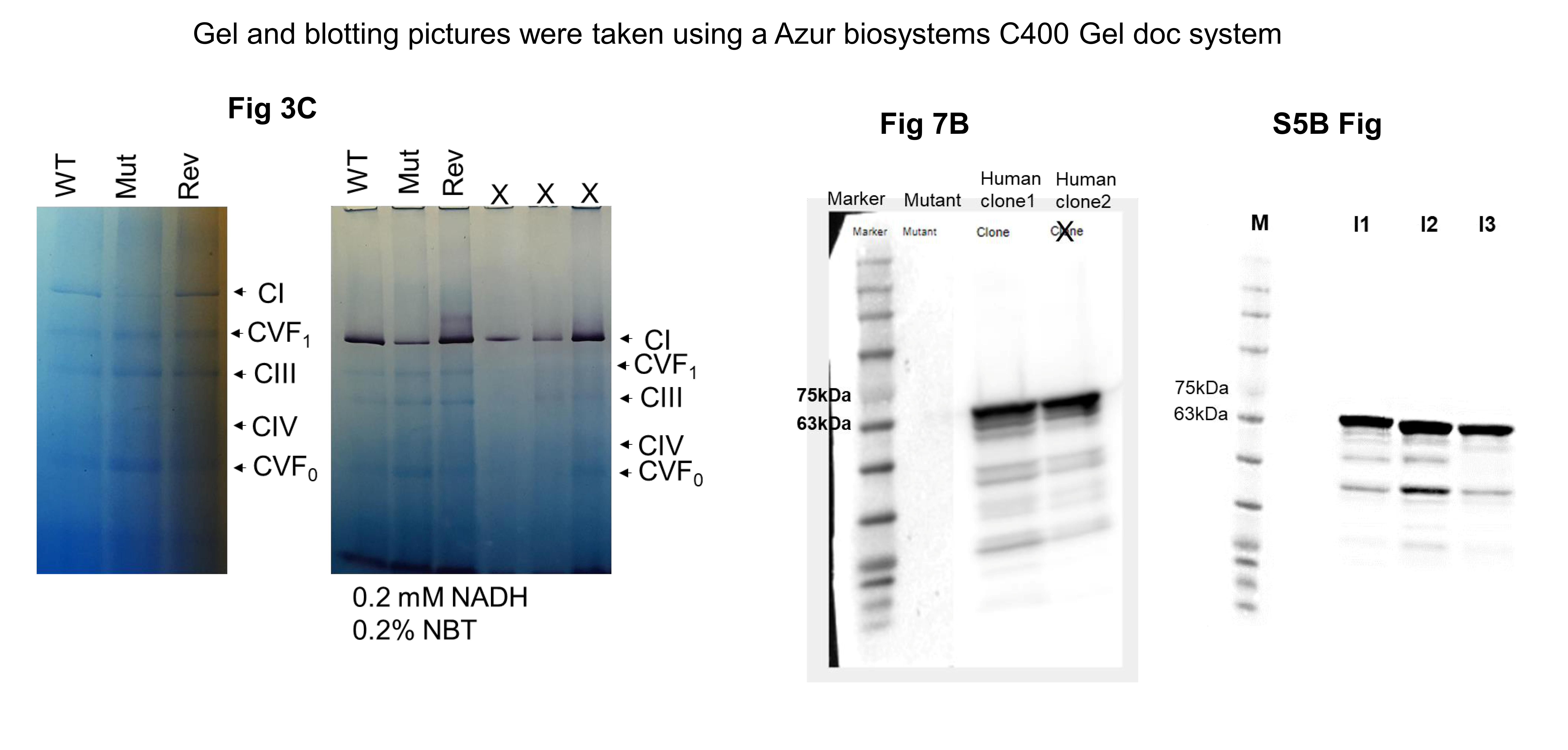

Supplement: S1 Raw Images — (TIFF) [file pbio.3000957.s008.tiff]
